# Development of thresholds and a visualization tool for use of a blood test in routine clinical dementia practice

**DOI:** 10.1002/alz.14088

**Published:** 2024-08-03

**Authors:** Inge M. W. Verberk, Jolien Jutte, Maurice Y. Kingma, Sinthujah Vigneswaran, Mariam M. T. E. E. Gouda, Marie‐Paule van Engelen, Daniel Alcolea, Javier Arranz, Juan Fortea, Alberto Lleó, Claire Chevalier, Moira Marizzoni, Elsmarieke M. van de Giessen, Afina W. Lemstra, Yolande A. L. Pijnenburg, Wiesje M. van der Flier, Anouk den Braber, David Wilson, Martijn C. Schut, Argonde C. van Harten, Charlotte E. Teunissen

**Affiliations:** ^1^ Neurochemistry Laboratory, Department of Laboratory Medicine Amsterdam UMC, Vrije Universiteit Amsterdam, Amsterdam Neuroscience Amsterdam The Netherlands; ^2^ Translational Artificial Intelligence Laboratory, Department of Laboratory Medicine Amsterdam UMC, Vrije Universiteit Amsterdam, Amsterdam Public Health Amsterdam The Netherlands; ^3^ Alzheimer Center, Department of Neurology Amsterdam UMC, Vrije Universiteit Amsterdam, Amsterdam Neuroscience Amsterdam The Netherlands; ^4^ Sant Pau Memory Unit, Department of Neurology Hospital de la Santa Creu i Sant Pau – Biomedical Research Institute Sant Pau (IIB Sant Pau), Universitat Autònoma de Barcelona Barcelona Spain; ^5^ Memory Centre, Division of Geriatrics and Rehabilitation University Hospitals of Geneva and University of Geneva Geneva Switzerland; ^6^ Laboratory of Biological Psychiatry IRCCS Istituto Centro San Giovanni di Dio Fatebenefratelli Brescia Italy; ^7^ Department of Radiology and Nuclear Medicine Amsterdam UMC, Vrije Universiteit Amsterdam, Amsterdam Neuroscience Amsterdam The Netherlands; ^8^ Amsterdam Public Health, Methodology & Digital Health Amsterdam UMC, Vrije Universiteit Amsterdam Amsterdam The Netherlands; ^9^ Biological Psychology Vrije Universiteit Amsterdam Amsterdam The Netherlands; ^10^ Quanterix corporation Billerica Massachusetts USA

**Keywords:** Alzheimer, biomarker, blood test, dementia, glial fibrillary acidic protein, neurofilament light, phosphorylated tau, plasma

## Abstract

**INTRODUCTION:**

We developed a multimarker blood test result interpretation tool for the clinical dementia practice, including phosphorylated (P‐)tau181, amyloid‐beta (Abeta)42/40, glial fibrillary acidic protein (GFAP), and neurofilament light (NfL).

**METHODS:**

We measured the plasma biomarkers with Simoa (*n* = 1199), applied LASSO regression for biomarker selection and receiver operating characteristics (ROC) analyses to determine diagnostic accuracy. We validated our findings in two independent cohorts and constructed a visualization approach.

**RESULTS:**

P‐tau181, GFAP, and NfL were selected. This combination had area under the curve (AUC) = 83% to identify amyloid positivity in pre‐dementia stages, AUC = 87%–89% to differentiate Alzheimer's or controls from frontotemporal dementia, AUC = 74%–76% to differentiate Alzheimer's or controls from dementia with Lewy bodies. Highly reproducible AUCs were obtained in independent cohorts. The resulting visualization tool includes UpSet plots to visualize the stand‐alone biomarker results and density plots to visualize the biomarker results combined.

**DISCUSSION:**

Our multimarker blood test interpretation tool is ready for testing in real‐world clinical dementia settings.

**Highlights:**

- We developed a multimarker blood test interpretation tool for clinical dementia practice.
- Our interpretation tool includes plasma biomarkers P‐tau, GFAP, and NfL.
- Our tool is particularly useful for Alzheimer's and frontotemporal dementia diagnosis.

## 1 BACKGROUND

Patients referred to memory clinics for dementia diagnosis are heterogeneous in their clinical presentations. Alzheimer's disease (AD) is the most common etiology among the neurocognitive disorders, with key pathological hallmarks amyloid‐beta (Abeta) and tau aggregation together with neurodegeneration and neuroinflammation.^1^, ^2^ Other common neurocognitive disorders leading to dementia include frontotemporal lobar degeneration (FTLD), mostly pathologically characterized by the accumulation of TAR DNA‐binding protein 43 (TDP‐43) or the microtubule‐associated protein tau,^3^ and dementia with Lewy bodies (DLB), pathologically hallmarked by alpha‐synuclein inclusions in neurons yet often presented with comorbid AD pathology.^4^ AD and other neurocognitive disorders leading to dementia usually have long pre‐dementia stages^5^, ^6^ during which neuropathological processes are ongoing but symptoms are absent or mild. Also, symptoms can be non‐specific for the type of neurocognitive disorder that is ongoing. The pre‐dementia stages when pathological load is not yet too advanced are hypothesized to be the prime window for intervention with disease‐modifying therapeutics.^7^ Indeed, promising trial results for anti‐amyloid disease‐modifying treatments have recently been obtained for patients with mild cognitive impairment (MCI) or early dementia due to AD.^8^, ^9^ Now, with the first generation of anti‐amyloid AD drugs entering the market, it becomes of utmost importance to increase access to tools for the establishment of timely biological AD diagnoses. As opposed to the current standard diagnostic tests, amyloid positron emission tomography (PET), magnetic resonance imaging (MRI), and cerebrospinal fluid (CSF) testing,^1^ a blood test would be the affordable, sustainable, practical, and scalable method needed to assist in timely AD diagnoses in the heterogeneous clinical dementia practice. In addition, a blood test is the preferred method for repeated patient testing. It is to be expected that therapeutic interventions for other causes of neurocognitive disorders will follow.

In recent years, much progress has been made in the development of blood tests for neurocognitive disorders,^10^, ^11^ driven by advances in high‐sensitivity laboratory methods. The plasma markers currently proposed for implementation in dementia care settings include Abeta42/40, phosphorylated (P)‐tau isoforms, glial fibrillary acidic protein (GFAP), and neurofilament light (NfL).^12^ These markers associate with AD‐specific cerebral Abeta and tau aggregation (Abeta42/40 and P‐tau isoforms), or with parallel and/or downstream biological processes of neurocognitive disorders such as reactive astrocytosis and neurodegeneration (GFAP and NfL, respectively).^10^, ^11^ Before we can proceed to widespread clinical use of a neurocognitive blood test, several research gaps are to be solved.^12^ Since the daily clinical dementia practice is highly heterogeneous, with patients presenting with different etiologies and in different syndromal stages of their disease, and given that neurocognitive disorders have complex pathophysiology, a single plasma marker is unlikely to optimally inform clinical decision‐making.^13^, ^14^, ^15^, ^16^, ^17^, ^18^ Hence, one of the research gaps to be solved prior to widespread clinical implementation of a neurocognitive blood test is to understand how to interpret a multimarker blood test result comprehensively in the heterogeneous routine clinical dementia practice, and to understand its usefulness for various clinical questions such as (early) AD, FTD, or DLB diagnosis or differential AD diagnosis.

We hypothesized that a combination of the current key plasma biomarkers Abeta42/40, P‐tau, GFAP, and NfL can aid in (early) AD, FTD, or DLB diagnosis and in differential diagnosis of AD versus FTD or DLB in the daily clinical dementia practice. In this study, we aimed to establish ready‐to‐use thresholds including a result visualization tool for clinical result interpretation of the multimarker neurocognitive blood test including Abeta42/40, P‐tau181, GFAP, and NfL for various clinical questions, which we subsequently aimed to validate in two independent clinical cohorts.

## 2 METHODS

### 2.1 Cohorts

#### 2.1.1 Development cohort: Amsterdam Dementia Cohort

From the Amsterdam Dementia Cohort^19^, ^20^ we selected 1199 individuals who had given written informed consent to use medical data and biomaterials for scientific research purposes, who had an ethylenediaminetetraacetic acid (EDTA) plasma sample stored in the biobank and were diagnosed within 6 months of their blood draw with subjective cognitive decline (SCD; *n* = 323), MCI (*n* = 283), AD‐dementia (*n* = 320), FTD (*n* = 162; 72 behavioral variant FTD, 13 right temporal variant FTD, 59 primary progressive aphasia, 14 FTD‐amyotrophic lateral sclerosis, 4 other), or DLB (*n* = 111). Additionally, information on amyloid status based on amyloid PET or through CSF measurement within 6 months of blood draw had to be available. The patients with AD‐dementia were by definition required to test positive on an amyloid PET scan or to have an abnormal CSF AD biomarker profile. The medical ethical committee of the VU University Medical Center approved the study (Ref: 2016.061, Ref: 2017.315), and the study was in accordance with the Helsinki Declaration of 1975.

All individuals underwent a standardized dementia diagnostic work‐up between 2003 and 2021. This consisted of neurological, physical, and neuropsychological evaluation, brain MRI, apolipoprotein E (APOE) genotyping (APOE ε4 carriage defined as having at least one ε4 allele), CSF AD biomarker analysis, and/or an amyloid PET scan. Clinical diagnosis was subsequently established in a multidisciplinary consensus meeting according to applicable criteria for MCI, AD‐dementia, FTD, or DLB.^1^, ^21^, ^22^, ^23^, ^24^, ^25^, ^26^, ^27^, ^28^ A label of SCD was assigned when clinical and cognitive testing was within normal limits, thus criteria for MCI and dementia were not met, and criteria for other neurological or psychiatric diseases potentially causing cognitive deficits were not met.^29^, ^30^, ^31^

**Research in context**

1. **Systematic Review**: The authors reviewed the literature using PubMed. Phosphorylated (P‐)tau, amyloid‐beta(Abeta)42/40, glial fibrillary acidic protein (GFAP) and neurofilament light (NfL) are the current blood‐based biomarkers suggested for implementation in clinical dementia practice. Interpreting a multimarker blood test in clinical contexts is challenging.
2. **Interpretation**: We show that a combination of P‐tau, GFAP, and NfL contributes to (early) Alzheimer's disease (AD), frontotemporal dementia (FTD), or dementia with Lewy bodies (DLB) diagnosis and to their differential diagnoses. To interpret this multimarker blood test result in the clinical dementia contexts, we constructed a result interpretation approach that includes a visualization for the stand‐alone biomarker results (UpSet plots) and for the biomarker results combined (density plots).
3. **Future Directions**: Our multimarker neurocognitive blood test visualization and interpretation tool is ready for testing in real‐world clinical dementia settings.

#### 2.1.2 Validation cohort: Geneva gMAD/COSCODE cohort

From the Memory Center of the Geneva University Hospitals, we selected 313 gMAD/COSCODE participants who signed written informed consent.^32^ Individuals were selected when an EDTA plasma sample was stored in the biobank, and information on amyloid status obtained with an amyloid PET scan or through CSF measurement was available within 1 year of the blood draw. Cognitively normal/SCD,^29^, ^31^ MCI,^24^ and dementia^25^ stages were defined based on the relevant diagnostic criteria at the initial clinical assessment. Sociodemographic variables such as age, sex, and years of education were collected for all participants. Global cognition was assessed by Mini‐Mental State Examination (MMSE) and was available for 305 out of 313 participants (97%) within 1 year of the plasma sampling. For 269 out of 313 participants (86%), the cognitive disease stage was assessed within 1 year of the plasma sampling (225 were non‐demented [56 volunteers recruited via advertising organized by the Geneva memory Center, 45 SCD, 120 MCI, 4 other psychiatric diseases], and 44 had dementia). The study was approved by the Geneva Ethics Committee (Ref: CCER_2016‐01346, Ref: CCER_2020_00403) and is conducted in accordance with the Helsinki Declaration of 1975.

#### 2.1.3 Validation cohort: Barcelona SPIN cohort

From the Sant Pau Memory Unit, Barcelona, we selected 240 participants from the SPIN cohort who signed written informed consent.^33^ The selection included individuals who were cognitively normal (*n* = 31), had MCI (*n* = 62), or had dementia due to AD (*n* = 23), FTD (*n* = 56), or DLB (*n* = 68). Additionally, information on amyloid status was available as well as Simoa plasma biomarker data on Abeta42/40, GFAP, NfL, and P‐tau181, which were measured within the context of the bPRIDE project (unpublished work; Neurochemistry Laboratory, Amsterdam). All individuals underwent standard physical and cognitive evaluation, MRI, general laboratory blood testing, and a lumbar puncture for CSF biomarker testing. The diagnosis was established based on the applicable clinical criteria.^29^, ^30^, ^31^ The SPIN study has been approved by the Hospital de la Santa Creu i Sant Pau local ethical committee and is conducted in accordance with the Helsinki Declaration of 1975.

### 2.2 CSF or PET‐based amyloid status

For the Amsterdam Dementia Cohort, amyloid status was based on amyloid PET images (*n* = 309) and when unavailable based on CSF P‐tau181 and Abeta42 (*n* = 886) measurements. For four participants, the amyloid status was determined elsewhere based on CSF analysis, which we derived from the patient files. Amyloid PET scans were performed using [18F]Florbetaben (*n* = 177), [18F]Florbetapir (*n* = 48), [18F]Flutemetamol (*n* = 24) or [11C]PIB (*n* = 60) tracers as part of standard clinical care or research; procedures are described in more detail elsewhere.^30^, ^34^, ^35^, ^36^, ^37^ Based on the presence of fibrillary amyloid pathology in the neocortex, scans were visually rated as positive (Aβ+) or negative (Aβ−) by a nuclear medicine physician according to company guidelines or for [11C]PiB according to previously published methods.^38^ CSF AD biomarkers Abeta42, P‐tau181, and t‐tau were measured with Innotest ELISAs (enzyme‐linked immunosorbent assays; Fuijirebio, Belgium) until December 2017 (*n* = 971) and with Elecsys assays (Roche Diagnostics GmbH, Germany) after (*n* = 148). Innotest Abeta42 values were adjusted for the drift in the measurements that occurred over the years.^39^ Subsequently, the Innotest P‐tau181/Abeta42 ratio was calculated, and a threshold of >0.06 was applied to dichotomize individuals as Aβ+ (Youden's index threshold was calculated with the R software pROC package in the total Amsterdam Dementia Cohort with available amyloid PET and CSF innotest biomarker results (*n* = 599); validity of use of the P‐tau181/Abeta42 ratio for Aβ+ was shown earlier^40^). The threshold for Elecsys P‐tau181/Abeta42 is >0.02.^41^ For value alignment between CSF assay types, we also transformed the Elecsys values into Innotest equivalents using published formulas.^42^

For the Geneva gMAD/COSCODE cohort, amyloid status was obtained within 1 year of the blood draw, and based on amyloid PET (*n* = 161) performed with [18F]florbetapir (*n* = 4) or [18F]Flutametamol (*n* = 156) (*n* = 1 with missing information on the used tracer) or based on CSF measurements (*n* = 152). Amyloid PET was conducted as described elsewhere^32^, ^43^, ^44^ and visually rated as positive or negative by a nuclear medicine physician. If a visual read was unavailable but centiloid values were calculated (*n* = 2), we applied the cutoff of >30 centiloids to define a positive amyloid PET status.^45^ CSF biomarkers were measured using Innotest (*n* = 81) or Lumipulse G00II (*n* = 71). For Innotest measurements, CSF Abeta42 < 880.5 pg/mL was considered amyloid positive.^43^ For Lumipulse G00II, in accordance with manufacturer's guidelines, the CSF Abeta42/40 ratio < 0.069 was considered amyloid positive, or when CSF Abeta40 was unavailable CSF Abeta42 < 725 was considered amyloid positive.

For the Barcelona SPIN cohort, amyloid status was based on CSF measurements. CSF biomarkers were measured using Lumipulse G600II, and CSF Abeta42/40 ratio <0.062 was considered amyloid positive.^46^

### 2.3 Blood biomarker analyses

For all three cohorts, non‐fasted EDTA blood plasma was collected through venipuncture. Samples were centrifuged after a maximum of 2 h, for 10 min at 1800 ×*g* at room temperature in Amsterdam, for 15 min at 1700 ×*g* at room temperature in Geneva and for 10 min at 2000 ×*g* at 4°C in Barcelona. EDTA plasma was aliquoted in ≤0.5 mL‐portions in polypropylene or low binding protein tubes, and stored at −80°C in the local biobanks until dry‐ice transportation to the Neurochemistry Laboratory Amsterdam for use.

Prior to analysis, samples were shortly thawed at room temperature and centrifuged at 10,000 ×*g* for 10 min. Subsequently, Abeta1‐42, Abeta1‐40, GFAP, NfL (first freeze‐thaw cycle) and P‐tau181 levels (second freeze‐thaw cycle) were measured on the Simoa HDx analyzer with the Simoa Neurology 4‐plex E Kit (N4PE; Quanterix, USA; all three cohorts) and the Simoa pTau181 V2 Kit (Quanterix; Amsterdam Dementia Cohort, Barcelona cohort) or the Simoa pTau181 V2.1 kit (Quanterix; Geneva cohort), according to manufacturer's instructions and with four‐times automated sample dilution. Samples were measured in singlicate with the N4PE kit and in duplicates with the pTau181 kits. Calibration curves and quality control samples were measured in duplicates with both kits. Quality control samples were included in all N4PE and P‐tau181 Simoa runs, showing good inter‐assay coefficients of variation of <15% for all markers in each cohort (more details in Table S1).

The plasma Simoa measurements of all three cohorts were performed within the Amsterdam University Medical Center, Neurochemistry Laboratory, but with different N4PE and P‐tau181 kit lots leading to different absolute values. To align the plasma biomarker values of the Geneva and Barcelona cohorts to the plasma biomarker values of the Amsterdam cohort, we re‐measured 32 Amsterdam Dementia Cohort samples with the kit lot numbers used for the Geneva cohort and 35 Amsterdam Dementia Cohort samples with the kit lot numbers used for the Barcelona cohort. With Passing Bablok regression comparing the re‐measured values to the original values, value transformation formulas were obtained (Figure S1) and applied.

### 2.4 Statistical analysis

Statistical analysis was performed and figures were constructed with Python version 3.11.5, using packages *pandas*, *numpy*, *sklearn*, and *upsetplot*. We considered *p* < 0.05 significant. Plasma NfL values were corrected for age using earlier published formulas.^47^ Baseline clinical characteristics of the cohorts were compared using chi‐squared tests with Bonferroni correction for categorical variables and Kruskal Wallis test with post‐hoc Dunn's testing with Bonferroni correction for continuous variables (*p*‐values that are significant without Bonferroni correction are indicated as well).

We identified six clinically relevant questions, focused on early (AD), FTD or DLB diagnosis and AD differential diagnosis, to which all subsequent data analyses were tailored: (1) identify AD among all subjects across all clinical stages (i.e., Aβ+ in the total cohort), (2) identify AD in the pre‐dementia stages (i.e., Aβ+ in the SCD and MCI set), (3) discriminate AD from FTD, (4) discriminate controls (Aβ− SCD) from FTD, (5) discriminate AD from DLB, and (6) discriminate controls from DLB.

We started with the Amsterdam Dementia Cohort. We applied LASSO regressions with 1000 bootstrap iterations and the penalty term alpha optimized toward a minimized mean squared error in 10‐fold cross validation (alphas: total cohort, Aβ+ vs. Aβ− = 0.014; SCD + MCI subset, Aβ+ vs. Aβ− = 0.018; AD vs. FTD = 0.019; AD vs. DLB = 0.011; FTD vs. control = 0.029; DLB vs. controls = 0.113) to select the optimal combination of the plasma biomarkers Abeta42/40, P‐tau181, GFAP and age‐corrected NfL for each clinical question. If a marker was selected in 100% of the iterations for at least one of the clinical questions, we included that marker in the diagnostic panel for all six clinical questions. For each clinical question, we calculated receiver operating characteristics‐areas under the curve (ROC‐AUCs) with 10‐fold internal cross validation for each LASSO‐selected marker separately as well as for the LASSO‐selected markers combined in logistic regression, to determine the diagnostic performance of the markers as stand‐alone markers as well as the diagnostic performance of the markers when interpreted in aggregation. For our result visualization tool, we combined two visualization approaches. (1) Youden's index thresholds for each clinical question for each of the markers were determined without internal cross‐validation. These thresholds were applied to the data to classify the plasma marker results as normal or abnormal, to subsequently construct UpSet plots.^48^ (2) Predicted values (probabilities) of logistic regression analyses to combine the LASSO‐selected markers were calculated for each clinical question without internal cross validation. These probabilities were used to determine probability thresholds at Youden's index, at 90% sensitivity and at 90% specificity. The probabilities were also used to construct smooth fitted density plots color coded for diagnostic group, including horizontal lines representing the three probability thresholds. The UpSet plots visualize the stand‐alone plasma biomarker results, and the density plots visualize the plasma biomarker results in aggregation. Data of new patients can be extrapolated on and interpreted according to these two plot types.

Our analyses were validated in two independent cohorts: the Geneva and the Barcelona cohorts. The clinical questions identification of Aβ+ versus Aβ− across the clinical continuum and in the pre‐dementia stages (CN + MCI) could be validated in both datasets. The clinical questions AD and controls versus FTD and DLB could only be validated in the Barcelona dataset. First, we compared cohort characteristics of the validation cohorts to our development cohort based on the method of Debray and colleagues^49^ to determine the degree of relatedness between the validation and development cohorts aiming to increase interpretability of the validation results. We applied the Debray method to each of the six clinical questions, by constructing a binary logistic regression model (membership model) to estimate the likelihood that an individual belongs to the development cohort or to the validation cohort. The models' discriminatory accuracy was evaluated with the ROC‐AUC score using 10‐fold internal cross‐validation and incorporated the LASSO‐selected plasma biomarkers and the outcome variable amyloid status (Aβ−, Aβ+) or diagnosis (SCD, MCI, AD− dementia, FTD and/or DLB, dependent on the clinical question and cohort). Low AUC scores represent a similar case mix (i.e., cohorts compare well on main characteristics) indicative of result reproducibility, while high AUC scores represent a different case mix (i.e., cohorts compare poorly on main characteristics) indicative of result transportability.

Next, we continued to validate our Amsterdam Dementia Cohort classification models in the independent validation cohorts, focused on the identified optimal combination of the plasma biomarkers when interpreted in aggregation (the logistic regression models including the markers simultaneously). We applied the Amsterdam Dementia Cohort models to the validation datasets Geneva and Barcelona to assess the Amsterdam Dementia Cohort models’ effectiveness in discerning individuals with varying risks of a positive outcome (discrimination) and the agreement between predicted and actual positive outcome rates (calibration) for each clinical question. Discrimination metrics included ROC‐AUC (c statistics), accuracy, sensitivity, specificity and confusion matrices. Repeated calibration for the plasma biomarkers interpreted in aggregation was judged by the calibration‐in‐the‐large (deviation between average predicted risk and actual risk in the cohort; perfect value = 0), calibration slope (correspondence between predicted risk and actual risk among individuals; perfect value = 1), and a calibration plot (match between predicted and actual risks throughout all predicted probabilities; plotted following the guidelines of the TRIPOD statement,^50^ additionally employing Loess smoothing for better visualization of consistency across all predicted risk levels), as recommended by Debray and colleagues.^49^ We additionally recalculated individual biomarker and probability thresholds to compare threshold transportability between the cohorts.

## 3 RESULTS

### 3.1 Amsterdam Dementia Cohort characteristics

For the development of our diagnostic thresholds and visualization tool, we included a total of 1199 individuals from the Amsterdam Dementia Cohort. See Table 1 for cohort demographics and clinical characteristics. In general, our SCD Aβ− individuals were the youngest, our MCI Aβ− and DLB groups had less females, APOE ε4 carriage was most common in the Aβ+ groups (SCD, MCI, AD‐dementia, but also in DLB), and MMSE was highest in the SCD groups followed by the MCI groups, and was lowest in the dementia groups (AD‐dementia, FTD, DLB) (*p*‐values for all demographic group comparisons are presented in Table S2).

**TABLE 1:** Demographics and clinical characteristics of the Amsterdam Dementia Cohort

	SCD Aβ−	SCD Aβ+	MCI Aβ−	MCI Aβ+	AD dementia	FTD	DLB
Parameter	*n* = 259	*n* = 64	*n* = 116	*n* = 167	*n* = 320	*n* = 162	*n* = 111
**Demographics**							
Age, years	59 ± 8	68 ± 6	63 ± 8	67 ± 7	64 ± 8	63 ± 9	69 ± 7
Female sex	108 (42)	35 (55)	23 (20)	72 (43)	186 (58)	76 (47)	19 (17)
APOEε4 carriage	73 (29)	47 (78)	31 (27)	127 (80)	208 (65)	45 (29)	60 (58)
MMSE	28 ± 1.6	28 ± 1.3	27 ± 2.3	26 ± 2.1	21 ± 4.4	23 ± 5.0	22 ± 5.0
Education, years	12 ± 3.0	13 ± 3.2	11 ± 3.4	12 ± 3.1	11 ± 2.8	11 ± 2.5	11 ± 2.9
**CSF markers**							
Abeta42, pg/mL	1126 ± 221	734 ± 222	1118 ± 253	633 ± 152	588 ± 102	1027 ± 344	811 ± 272
P‐tau181, pg/mL	43 ± 17	76 ± 31	45 ± 15	82 ± 30	95 ± 35	49 ± 21	53 ± 25
T‐tau, pg/mL	258 ± 141	579 ± 374	278 ± 125	609 ± 288	799 ± 374	420 ± 202	383 ± 254
**Plasma markers**							
Abeta42/40	0.06 ± 0.02	0.05 ± 0.01	0.06 ± 0.01	0.05 ± 0.01	0.05 ± 0.01	0.06 ± 0.01	0.05 ± 0.01
P‐tau181, pg/mL	1.44 ± 0.88	2.30 ± 1.01	1.53 ± 0.79	2.39 ± 1.16	2.81 ± 1.08	1.75 ± 1.45	2.00 ± 1.25
GFAP, pg/mL	66.0 ± 36	124 ± 66	73.6 ± 34	116 ± 50	145 ± 70	103 ± 59	118 ± 72
NfL, pg/mL	11.4 ± 6.7	17.8 ± 6.7	15.6 ± 13	17.7 ± 9.9	20.3 ± 12	38.3 ± 38	22.8 ± 21

*Notes*: Data are presented as mean ± SD or *n* (%). APOE ε4 carriage is defined when an individual carries at least one APOE ε4 allele. Elecsys CSF values were transformed into their Innotest equivalents according to published formulas^42^ (true for *n* = 148). Amyloid status was according to amyloid positron emission tomography (*n* = 309), cerebrospinal fluid P‐tau181/Abeta42 ratio, measured with Innotest (*n* = 741), Elecsys (*n* = 113) or a combination of Innotest and Elecsys (*n* = 32), or taken from the patient files (*n* = 4). CSF Abeta42 and CSF P‐tau181 were missing for *n* = 80, CSF T‐tau was missing for *n* = 82, MMSE was missing for *n* = 8, APOE ε4 carriage was missing for *n* = 42, plasma Abeta42/40, GFAP and NfL were missing for *n* = 2, plasma p‐tau181 was missing for *n* = 15.

Abbreviations: Abeta, amyloid beta; AD, Alzheimer's disease; APOE, apolipoprotein E; DLB, dementia with Lewy bodies; FTD, frontotemporal dementia; GFAP, glial fibrillary acidic protein; MCI, mild cognitive impairment; MMSE, Mini‐Mental State Examination; NfL, neurofilament light; P‐tau, phosphorylated tau; SCD, subjective cognitive decline; T‐tau = total tau.

**FIGURE 1:**
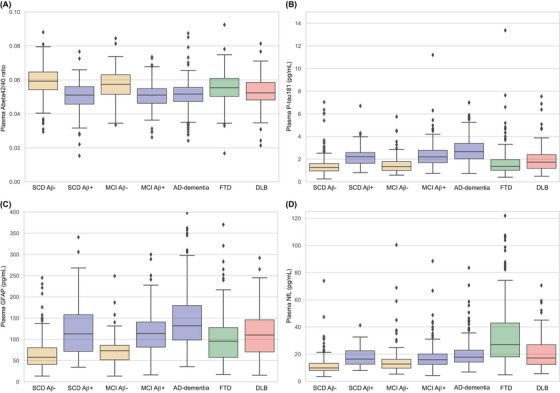
Boxplots of plasma biomarkers according to their diagnostic group in the Amsterdam Dementia Cohort. Aβ, amyloid status; Abeta, amyloid beta; AD, Alzheimer's disease; DLB, dementia with Lewy bodies; FTD, frontotemporal dementia; GFAP, glial fibrillary acidic protein; MCI, mild cognitive impairment; NfL, neurofilament light; P‐tau, phosphorylated tau; SCD, subjective cognitive decline.

Boxplots of the plasma markers Abeta42/40, P‐tau181, GFAP, and NfL per diagnostic group are shown in Figure 1 (*p*‐values for all plasma marker group comparisons are presented in Table S2; individual data point boxplots are shown in Figure S2). In general, Abeta42/40 levels were lower in the Aβ+ groups (SCD, MCI, AD‐dementia) and also in the DLB group compared to the Aβ− groups (SCD, MCI). In general, P‐tau181 and GFAP showed the opposite, levels were higher in the Aβ+ groups (SCD, MCI, AD‐dementia) compared to the Aβ− groups (SCD, MCI). For GFAP, levels were also higher in the FTD and DLB groups compared to the Aβ− groups (SCD, MCI), while for P‐tau181 levels were higher in the DLB group but not in the FTD group compared to the Aβ− groups (SCD, MCI). NfL levels were lowest in the SCD Aβ− group and highest in the FTD group.

### 3.2 Diagnostic plasma marker panel selection and its predictive accuracy for clinically relevant questions

Upon subsetting our Amsterdam Dementia Cohort sample according to our six a priori defined clinically relevant questions (Aβ+ in the total cohort, Aβ+ in the SCD and MCI subset, AD vs. FTD, controls vs. FTD, AD vs. DLB, controls vs. DLB) and applying the LASSO regression with 1000 iterations, we found that plasma P‐tau181, GFAP and (age‐corrected) NfL are the relevant diagnostic biomarkers while Abeta42/40 did not have additional diagnostic value (Table 2). P‐tau181 and GFAP were most robustly selected (P‐tau181 in 100% of the iterations for all six questions, GFAP in100% of the iterations for five of the six questions and in 98% of the iterations for the other question), followed by NfL (in 100% of the iterations for four of the six questions, in 17% and 0% of the iterations for the other two questions).

**TABLE 2:** Frequency of plasma biomarker selection in LASSO regression with 1000 bootstrap iterations for six clinical questions in the Amsterdam Dementia Cohort

Parameter	Abeta42/40	P‐tau181	GFAP	Age‐corrected NfL
1. Aβ+/Aβ− in total cohort	0%	100%	100%	100%
2. Aβ+/Aβ− in SCD + MCI	0%	100%	100%	0%
3. AD vs. FTD	0%	100%	100%	100%
4. Controls vs. FTD	0%	100%	100%	100%
5. AD vs. DLB	0%	100%	98%	17%
6. Controls vs. DLB	0%	100%	100%	100%

*Notes*: For the six clinical questions, % of a biomarker being selected within the 1000 iterations is presented. We applied LASSO regression with 1000 bootstrap iterations where the penalty terms were optimized toward a minimized mean squared error in 10‐fold cross validation.

Abbreviations: Aβ−, negative amyloid status; Aβ+, positive amyloid status; Abeta, amyloid beta; AD, Alzheimer's disease; DLB, dementia with Lewy bodies; FTD, frontotemporal dementia; GFAP, glial fibrillary acidic protein; MCI, mild cognitive impairment; NfL, neurofilament light; P‐tau, phosphorylated tau; SCD, subjective cognitive decline.

**FIGURE 2:**
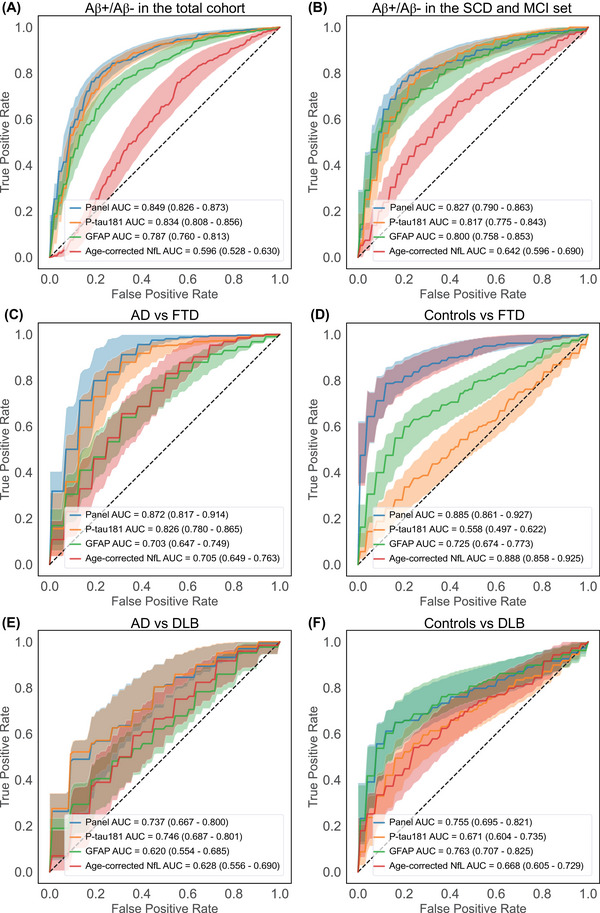
ROC curves with AUCs for the LASSO‐selected markers P‐tau181, GFAP, and NfL and their combination into a panel in the Amsterdam Dementia Cohort. ROC AUCs with 95% confidence intervals were computed with internal 10‐fold cross validation, for the clinical questions amyloid negative status (Aβ−) versus amyloid positive status (Aβ+) in the total cohort (A), Aβ− versus Aβ+ in the SCD and MCI set (B), AD versus FTD (C), controls versus FTD (D), AD versus DLB (E) and controls versus DLB (F). The panel consists of P‐tau181, GFAP, and age‐corrected NfL. NfL was corrected for age using a published formula.^47^ AD, Alzheimer's disease; AUC, area under the curve; DLB, dementia with Lewy bodies; FTD, frontotemporal dementia; GFAP, glial fibrillary acidic protein; MCI, mild cognitive impairment; NfL, neurofilament light; P‐tau, phosphorylated tau; ROC, receiver operating characteristics; SCD, subjective cognitive decline.

Subsequent ROC analysis with internal 10‐fold cross‐validation to determine the diagnostic accuracy of the clinically relevant plasma markers P‐tau181, GFAP, and NfL as stand‐alone markers showed AUCs ranging from poor to good dependent on the clinical question asked (ROC curves with AUCs presented in Figure 2). P‐tau181 had particularly high AUCs for identification of amyloid status (total cohort: 0.83, 95%CI: 0.81–0.86; SCD + MCI subset: AUC = 0.82, 95% CI: 0.78–0.84) and to discriminate AD from FTD (AUC = 0.83, 95%CI: 0.78–0.87). GFAP had good AUCs for identification of amyloid status (total cohort: AUC = 0.79, 95%CI: 0.76–0.81; SCD and MCI set: AUC = 0.80, 95%CI: 0.76–0.85), and reasonable AUCs for discrimination of controls from FTD (AUC = 0.73, 95%CI: 0.67–0.77) or controls from DLB (AUC = 0.76, 95%CI: 0.71–0.83). NfL had a particularly high AUC for discrimination of controls from FTD (AUC = 0.89, 95%CI: 0.86–0.93).

ROC‐AUCs computed with internal 10‐fold cross‐validation for the LASSO‐selected markers combined ranged from AUC = 0.74–0.85 dependent on the clinical question asked (ROC curves and AUCs presented in Figure 2). The three biomarkers interpreted in aggregation performed well for the identification of amyloid status and to discriminate FTD from AD and controls (AUCs ≥ 0.83), but weaker for the questions AD or controls versus DLB (AUCs ≤ 0.76). The three biomarkers interpreted in aggregation had visually higher ROC‐AUCs than the best‐performing stand‐alone biomarker for the identification of amyloid status in the total cohort and the SCD and MCI set (ΔAUCs ≥ 0.01), and for differentiating AD from FTD (ΔAUCs = 0.05), while the aggregate did not improve the differentiation of controls from FTD, or the differentiation of AD or controls from DLB (Figure 2).[Fig alz14088-fig-0003]

### 3.3 Development of the clinically implementable blood test result interpretation tool

Following the LASSO and ROC‐AUC analyses, we developed two visualizations to be combined into one clinically implementable plasma marker result visualization tool to aid in the interpretation of individual patient results: (1) UpSet plots to visualize the proportion of patients that score normal/abnormal on a specific combination of plasma biomarker results according to a diagnostic group and (2) density plots to visualize the likelihood a patient with a certain probability score based on the plasma biomarker results interpreted in aggregation belongs to a certain diagnostic group. The plots are specific to each clinical question. The results on which the plots are based are detailed below.

#### 3.3.1 UpSet plots

**FIGURE 3 alz14088-fig-0003:**
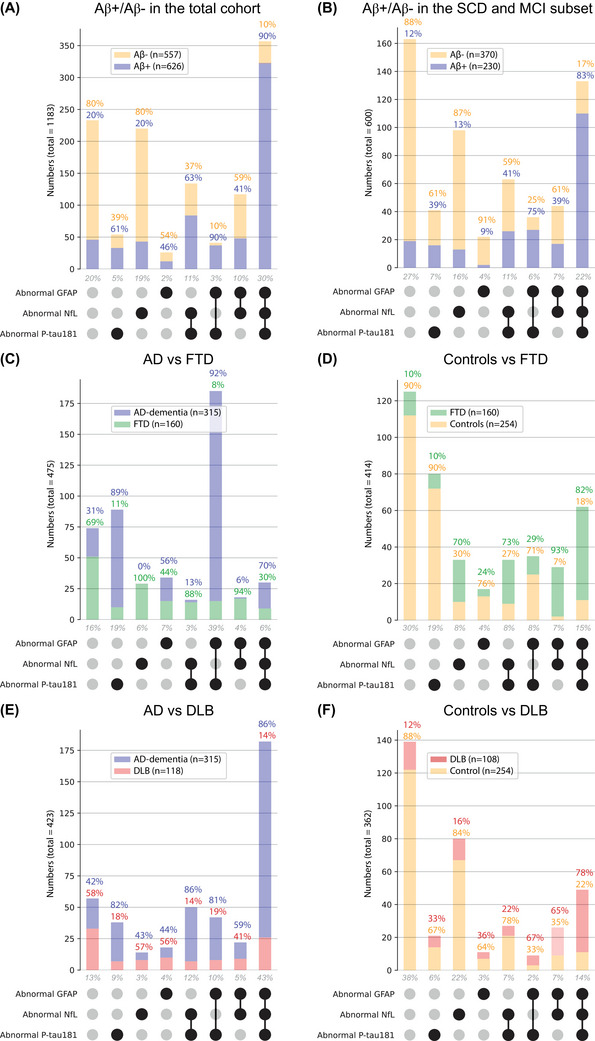
UpSet plots visualizing the proportion of Amsterdam Dementia Cohort individuals with certain combinations of normal/abnormal plasma biomarkers according to their diagnostic group for the six clinical questions. Participants were normal (gray dot) or abnormal (black dot) on their plasma biomarker results after applying Youden's index thresholds computed from receiver operating characteristics curves plotted for each clinical question. The proportion of participants with a certain combination of normal/abnormal plasma biomarker results according to their diagnostic groups and for the clinical questions amyloid negative status (Aβ−) versus amyloid positive status (Aβ+) in the total cohort (A), Aβ− versus Aβ+ in the SCD and MCI set (B), AD versus FTD (C), controls versus FTD (D), AD versus DLB (E) and controls versus DLB (F) are visualized. AD, Alzheimer's disease; DLB, dementia with Lewy bodies; FTD, frontotemporal dementia; GFAP, glial fibrillary acidic protein; MCI, mild cognitive impairment; NfL, neurofilament light (age‐corrected); P‐tau, phosphorylated tau; SCD, subjective cognitive decline.

Youden's index thresholds used to construct the UpSet plots for each LASSO‐selected marker for each clinical question are presented in Table S3. The UpSet plots for each clinical question are presented in Figure 3.

The UpSet plots for the identification of amyloid status show that the largest proportions of our study cohort had either three normal plasma marker results (total cohort: 20%; SCD + MCI subset: 27%), where most participants were indeed Aβ− (total cohort: 80% Aβ−; SCD + MCI set: 88% Aβ−), or had three abnormal plasma marker results (total cohort: 30%; SCD + MCI set: 22%), where most participants were indeed Aβ+ (total cohort: 90% Aβ+; SCD + MCI set: 83% Aβ−). Another common category was normal P‐tau181 and GFAP results in combination with abnormal NfL results (total cohort: 19%; SCD + MCI set: 16%). This result was highly indicative of Aβ− (total cohort: 80% Aβ−; SCD + MCI set: 87% Aβ−). The opposite, abnormal P‐tau181 and GFAP with normal NfL results was less common (total cohort: 3%; SCD + MCI set: 7%), but this result is highly indicative of Aβ+ (total cohort: 90% Aβ+; SCD + MCI set: 75% Aβ+).

The UpSet plot for AD versus FTD shows that a combination of abnormal P‐tau181 and GFAP with normal NfL (39% with this result, 92% had AD) or abnormal P‐tau181 with normal GFAP and NfL (19% with this result, 89% AD) are highly indicative of AD, while having normal P‐tau181 but abnormal NfL combined with either normal GFAP (6% with this result, 100% FTD) or abnormal GFAP (4% with this result, 94% FTD) are highly indicative of FTD. The UpSet plot for controls versus FTD also shows that abnormal NfL is highly indicative of FTD, with high prevalence of FTD in the groups having an abnormal NfL result in combination with normal or abnormal result for the other two plasma markers (7%–15% with this result, 70%–93% FTD). In contrast, there is a high prevalence of controls in the groups with normal NfL results in combination with either normal or abnormal results on the other two biomarkers (4%–30%, 71%–90% controls).

The UpSet plot visualizing AD versus DLB shows no specific combination of normal/abnormal plasma marker results that is highly indicative of DLB. AD versus DLB prevalences are approximately equal for most of the combinations of normal/abnormal plasma marker results. The UpSet plot visualizing controls versus DLB indicates that abnormal GFAP in combination with either or both abnormal P‐tau181 and NfL is somewhat indicative of DLB (scored by 2%–14%, 67%–78% DLB).

#### 3.3.2 Density plots

**FIGURE 4:**
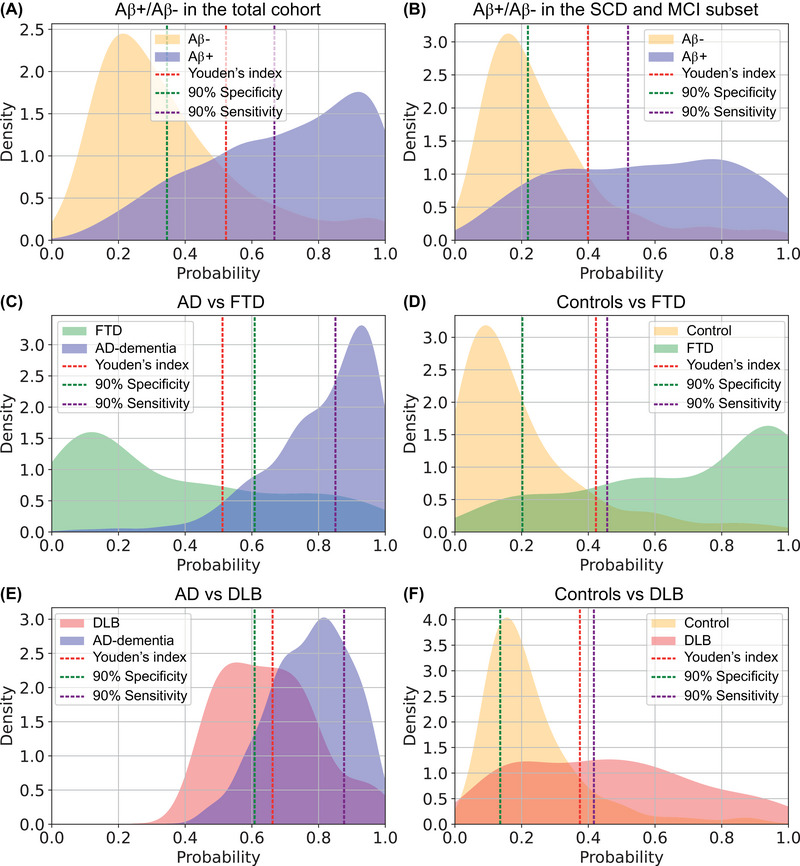
Density plots visualizing the discriminative accuracy of P‐tau181, GFAP, and age‐corrected NfL interpreted in aggregation in the Amsterdam Dementia Cohort for six clinical questions. Probabilities of logistic regression analysis combining the LASSO‐selected markers P‐tau181, GFAP, and age‐corrected NfL were plotted and color coded for diagnostic group, including probability threshold lines at the Youden's index, at 90% specificity and at 90% sensitivity, according to their diagnostic groups and for the clinical questions amyloid negative status (Aβ−) versus amyloid positive status (Aβ+) in the total cohort (A), Aβ− versus Aβ+ in the SCD and MCI set (B), AD versus FTD (C), controls versus FTD (D), AD versus DLB (E), and controls versus DLB (F). Continuous probability density curves were applied to represent the probability thresholds for smoothed fitting. NfL was corrected for age using a published formula.^47^ AD, Alzheimer's disease; DLB, dementia with Lewy bodies; FTD, frontotemporal dementia; GFAP, glial fibrillary acidic protein; MCI, mild cognitive impairment; NfL, neurofilament light; P‐tau181, phosphorylated tau181; SCD, subjective cognitive decline.

The logistic regression probability thresholds at the Youden's index and at 90% sensitivity and 90% specificity for the combination of the LASSO‐selected markers interpreted in aggregation for each clinical question are presented in Table S4 (linear predictor formulas presented in Table S5). The constructed density plots are presented in Figure 4, visualizing the proportions of our study cohort with certain probability levels according to their diagnostic group. We classified the probabilities lower than the 90% specificity thresholds and higher than 90% sensitivity thresholds as low and high likelihood group respectively, and the probabilities falling between these thresholds as undetermined or intermediate likelihood. Table S6 shows the proportions of our individuals falling in the low, undetermined/intermediate and high likelihood groups according to their diagnosis.

The density plots show that the combination of P‐tau181, GFAP and age‐corrected NfL when interpreted in aggregation performs well to differentiate Aβ− from Aβ+ in the total cohort and in the SCD and MCI subset and to discriminate FTD from AD or from controls. For these four clinical questions, large proportions of individuals fell into the low likelihood (range 25%–45%) and high likelihood groups (range 28%–36%). Within those low and high likelihood groups, there was also a high level of agreement with diagnosis (range: 79%–92%). The proportions of individuals falling into the undetermined/intermediate likelihood groups for these four clinical questions ranged from 20% to 35%.

The density plots to discriminate DLB from AD or from controls visualize poorer discriminative accuracy for the biomarker panel P‐tau181, GFAP, and age‐corrected NfL when interpreted in aggregation, with greater proportions of individuals with probabilities falling into the undetermined/intermediate likelihood group (62% and 56%, respectively), and with more individuals that are incorrectly diagnosed within the low (41% and 15%, respectively) and high likelihood groups (12% and 29%, respectively).

#### 3.3.3 Individual patient examples interpreted according to the blood test result interpretation tool

**FIGURE 5:**
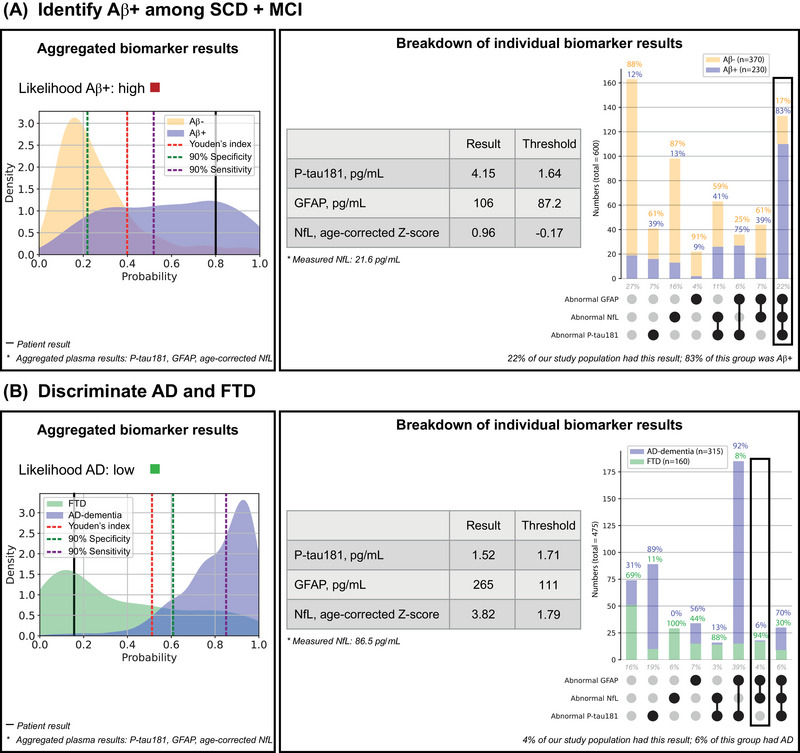
Clinically implementable plasma result visualization tool. This figure presents two example results of individuals of our cohort: (A) male with MCI, age 65 years, and biomarker values of 4.15 pg/mL P‐tau181, 106 pg/mL GFAP and 21.8 pg/mL NfL; (B) female with FTD, age 65 years, and biomarker values of 1.52 pg/mL P‐tau181, 265 pg/mL GFAP, and 86.5 pg/mL NfL. These examples illustrate how the combination of the density plot, the UpSet plot, and the table with biomarker values together with their thresholds can be used for clinical interpretation of plasma biomarker results of new patients presenting at the memory clinic. FTD, frontotemporal dementia; MCI, mild cognitive impairment; NfL, neurofilament light; P‐tau181, phosphorylated tau181.

Two individual patient results to exemplify how the blood test result visualization tool is to be used for clinical interpretation of plasma biomarker results of new patients presenting at the memory clinic are presented in Figure 5. The combined interface includes the UpSet plot and density plot as well as a table to enter individual biomarker results to compare them against their thresholds, and is specific to each clinical question so the medical doctor can choose which clinical question is applicable to his or her patient. In Figure 5, two individual patient results according to two of the six developed interfaces are presented: an Aβ+ male with MCI, age 65 years (Figure 5A), and a female with FTD, age 65 years (Figure 5B).

To visualize the individual patient results in the interface, a probability level is calculated by filling out the logistic regression predictor formulas (Table S5) using the patient's plasma marker levels and plotted as a vertical line in the density plot. In addition, a box is drawn in the UpSet plots to visualize in which group the patient falls according to their combination of normal/abnormal results on the three plasma markers.

### 3.4 External validation in the Geneva and Barcelona cohorts

#### 3.4.1 Validation cohort characteristics in comparison to the Amsterdam Dementia Cohort

Finally, we validated our Amsterdam Dementia Cohort classification models in two independent cohorts: the Geneva cohort (*n* = 313; detailed cohort characteristics in Tables S7 and S8) and the Barcelona cohort (*n* = 240; detailed cohort characteristics in Tables S9 and S10).

The Debray membership models including P‐tau181, GFAP and age‐corrected NfL in addition to amyloid status or diagnosis (dependent on the clinical question asked) showed ROC‐AUCs of 0.65–0.80 (lowest AUC: Barcelona vs. Amsterdam Dementia Cohort, clinical question Aβ+/− in the total cohort; highest AUC: Barcelona vs. Amsterdam Dementia Cohort, clinical question controls vs. FTD; Table S11), indicative of the validation cohorts having a different case mix (i.e., characteristics compare poorly) than the Amsterdam Dementia Cohort, explaining both validation cohorts are useful to assess result transportability rather than reproducibility.

#### 3.4.2 Validation of the Amsterdam Dementia Cohort classification models

The classification model validation focused on the P‐tau181, GFAP, and age‐corrected NfL interpretation in aggregation, applying the logistic regression models developed in the Amsterdam Dementia Cohort for the six clinical questions to the same six clinical questions asked in the validation cohorts. We obtained highly reproducible AUCs in the Geneva cohort (AUCs = 0.84 and 0.82; compared to AUCs = 0.85 and 0.83 in the Amsterdam Dementia Cohort; Table S12) and also reproducible AUCs in the Barcelona cohort (range AUCs = 0.63–0.92; compared to the Amsterdam Dementia Cohort: range AUCs = 0.75–0.90; Table S12). Overall, AUCs were most robustly reproduced for prediction of Aβ+ status in the total cohort and the cognitively normal/SCD and MCI set and to discriminate FTD from AD (≤0.05 change in AUC). Notably, AUCs were higher in the Barcelona cohort compared to the Amsterdam Dementia Cohort for the clinical questions FTD versus AD and controls versus DLB (ΔAUCs ≥ 0.05). Additional validation metrics are presented in the supplement (accuracy, sensitivity, and specificity: Table S13; confusion matrices: Table S14). The repeated calibration and predicted probability plots (Figure S3 and S4) show relatively good calibration but with some overestimation of the model's predicted probabilities compared to the actual probabilities for the clinical questions Aβ+ versus Aβ− in the total cohort an in the cognitively normal/SCD and MCI set, in both validation datasets (calibration‐in‐the‐large scores range: −0.01–0.24; Table S12). Calibration is poorer for the other clinical questions, with a large overestimation of predicted versus actual probabilities for the models AD versus FTD (calibration‐in‐the‐large score of 0.38) and AD versus DLB (calibration‐in‐the‐large score of 0.55), and a large underestimation of predicted versus actual probabilities for the models control versus FTD and control versus DLB (calibration‐in‐the‐large score of −0.32 and −0.28, respectively). In agreement, recalculation of Youden's index thresholds for the individual biomarkers in the validation datasets resulted in cohort‐specific thresholds (Table S15).

## 4 DISCUSSION

We developed and validated a clinically applicable blood test result visualization tool to facilitate the implementation of the multimarker blood test for neurocognitive disorders in routine clinical dementia practice. We showed that, among the current core plasma biomarkers, a combination of plasma P‐tau, GFAP, and NfL is informative for clinically relevant questions: (early) AD, FTD, or DLB (differential) diagnosis. We showed that particularly AD and FTD can be identified with this neurocognitive blood test. Strong potential of plasma P‐tau181, GFAP, and NfL as biomarkers for AD and for differentiation of AD from FTD was recently confirmed in an unselected clinical cohort,^51^ indicating the potential of our neurocognitive blood test visualization tool to be informative in the real‐world clinical setting. Our validated neurocognitive blood test tool facilitates the interpretation of the plasma biomarker results as stand‐alone results and as an aggregated result score, providing comprehensive and complementary information to the end users.

We guided our analyses by clinically relevant questions, which were decided upon during brainstorm sessions with neurologists who were envisioned as the first end‐users of our study results. Firstly, our tool is developed to inform on the likelihood of a memory clinic patient to have an abnormal amyloid status. Providing a biological AD diagnosis with an accessible blood test is highly relevant in the current era of anti‐amyloid treatments coming to age, to enable timely diagnoses to decide who to treat. Additionally, we developed our tool to inform on the likelihood that an individual's cognitive impairment is not caused by AD but by another common form of dementia. Patients with clinical FTD or DLB can have AD co‐pathology (i.e., Aβ+ status), however these patients will initially not be treated with anti‐amyloid treatments as first real‐world experience and confidence in the medications should be gained in patients with purer forms of AD. In addition, an FTD or DLB diagnosis requires their own symptomatic treatment and care plan. Discriminating different forms of dementia is thus important. Last, we developed our tool to inform on the likelihood a patient suffers from a common neurocognitive disease causing dementia, or from something else causing their cognitive worries. This could be for example a psychiatric illness, although this disease group was not specifically included in our dataset. Discriminating neurocognitive diseases from other causes of cognitive worries is important in light of prescreening individuals with cognitive complaints. This could lead to only referring a subset of individuals to highly specialized memory clinics, which ultimately increases access to and lowering the burden on the health care systems.

Multiple studies investigated the potential of the biomarkers Abeta42/40, P‐tau, GFAP, and NfL as stand‐alone biomarkers or interpreted in aggregation to inform on the diagnosis of neurocognitive disorders.^13^, ^14^, ^15^, ^16^, ^52^, ^53^ In line with those studies, we found that a combination of biomarkers leads to slightly better patient classification compared to the use of a single biomarker.^13^, ^15^, ^18^ Our results also showed in agreement with a recent study^52^ that the current version of the neurocognitive blood test including P‐tau, GFAP, and NfL is less useful to discriminate AD from DLB, which is in line with expectations due to high prevalence of AD co‐pathology in DLB (in the current dataset: 49%). Although diagnostically challenging due to overlapping presentations between DLB and AD, DLB‐specific clinical features together with cognitive tasks seems currently most informative for DLB differential diagnosis.^54^ The diagnostic performance for FTD was much better than for DLB in our study, due to NfL being a strong biomarker for the most common types of FTD as was also observed by others,^55^, ^56^ and because there is a lower prevalence of AD co‐pathology (in the current dataset: 17%) leading to higher specificity of especially P‐tau181 for AD.^57^ Especially as visualized in our UpSet plots, we utilize the combination of P‐tau181 negativity with NfL positivity to inform on an FTD diagnosis, strengthening the diagnostic value of these two biomarkers when interpreted in combination. There is need of development of novel blood‐based biomarkers specifically for synucleinopathies and TDP‐43, 3R tauopathies, and 4R tauopathies, to enhance the clinical performance of a neurocognitive blood test for FTD and DLB. Our tool can be updated with such novel, disease‐specific markers when these come available.

In contrast to many previous studies, our neurocognitive blood test visualization tool does not include specific AD or more general neurocognitive disorders‐related risk factors to enhance the diagnostic accuracy. Risk factors often included in blood test classification models are APOE ε4 carriage, age, sex, and/or a cognitive performance score (e.g., MMSE). For example, in the PrecivityAD mass spectrometry blood test, APOE is included to enhance the predictive performance^58^ We decided against inclusion of risk factors, as we sought to reflect a current biological state of patients. In line with this approach, interpretation of individual patient results of the core Alzheimer's CSF or PET biomarkers in clinical dementia practice is also based on the stand‐alone biomarker results. Medical doctors judge the biomarker results together with the clinical presentation of the patient and results of other diagnostic tests to get a comprehensive clinical picture of the individual patient. It is to note that data‐driven disease classifiers including clinical, molecular, cognitive, and imaging data have also been developed and were found clinically useful in diagnostic decision making.^59^ That being said, we did correct the plasma NfL levels for age in our study given its well‐recognized, strong age‐dependency^47^, ^60^ that also remains within disease groups,^47^ making this marker improper to interpret without taking age into account. Some studies suggest that certain AD plasma biomarkers might also need correction for presence of specific comorbidities or clinical characteristics.^61^, ^62^, ^63^, ^64^ When consistent results on clinical impact of correcting plasma biomarker levels for comorbidities and clinical characteristics are being obtained, we can update our neurocognitive blood test tool accordingly.

Strengths of our study include the clinical translational nature of our study. Also, the thorough model development in a large clinical cohort is a strength, and that we validated our findings in two external validation cohorts. It is to note that our external validation showed good reproducibility of AUCs, though location‐specific threshold might still be needed until full harmonization between centers is complete. Our Debray comparison^49^ of the cohort characteristics highlighted the differences between the cohorts, potentially explaining the need for location‐specific thresholds. Among the limitations of the study is the use of P‐tau181 instead of P‐tau217, although we still obtained acceptable diagnostic accuracies. Literature is consistently showing that P‐tau217 is a stronger biomarker for AD than P‐tau181,^65^ and regulatory approval (In Vitro Diagnostic Medical Devices Regulation [IVDR], Clinical Laboratory Improvement Amendments [CLIA], US Food and Drug Administration [FDA]) of commercially available immunoassays for P‐tau217 is expected in due time. We used the Simoa P‐tau181 test as at time of study conceptualization and laboratory measurements this test was logistically the only commercially available plasma P‐tau test. Reliable access to and a steady supply chain of an analytical assay is essential when aiming for clinical implementation of tests. In future research, we will assess if replacing P‐tau181 by P‐tau217 improves the diagnostic accuracy of our models. Within that effort, we will re‐evaluate what the optimal clinical panel would be using various feature selection methods. Another limitation of our study is the selection bias we introduced by focusing on specific diagnostic groups and by selecting based on availability of an amyloid status and plasma samples. We specifically excluded individuals suffering from other, common as well as less common forms of dementia in the development and validation of our models, for example, vascular or mixed dementia, while those patient groups would also benefit from a blood test to assist in diagnostic decision making. A next step is the prospective evaluation of the here developed and validated neurocognitive blood test result visualization tool in daily clinical practice without any patient selection, which we recently initiated. Such a prospective study in an unselected cohort will also inform on how to handle inconclusive results, for example, when our density plots visualize an undetermined/intermediate likelihood for an expected diagnosis and the UpSet plots visualize an inconclusive or very low prevalent combination of positive/negative plasma marker outcomes. It has been suggested that in such cases a two‐step workflow with screening with a neurocognitive blood test and subsequent confirmatory testing using CSF or with PET in only uncertain cases might be a solution.^66^ Another next step is to assess the use of fully automated random access technologies for the biomarker measurements to improve overall access to the biomarker measurements and to evaluate cost‐effectiveness of single‐sample analysis. Potentially, our neurocognitive blood test visualization tool could be adapted to results obtained with other high‐sensitivity laboratory test for the markers P‐tau, GFAP, and NfL, to make our tool useful for other laboratories that use other immunoassay platforms. Also, the potential added value of Abeta42/40 could be re‐assessed after measuring it with other high‐performing automated assays.^67^, ^68^, ^69^, ^70^, ^71^, ^72^ The difference in plasma Abeta42/40 ratio between Aβ+ and Aβ− is small, earlier estimated at 6%–13% dependent on the technology used.^72^ Therefore analytical noise greatly affects the diagnostic accuracy of a plasma Abeta42/40 test,^72^ which a high‐performing fully automated assay might be able to overcome.

In conclusion, we successfully developed and validated a result visualization and interpretation tool for a neurocognitive blood test including the markers P‐tau181, GFAP, and NfL, which is ready for testing in the real‐world clinical dementia settings. Having a reliable result interpretation tool is one of the key steps to facilitate use of the blood test in the routine clinical dementia practice, as interpretation of a multimarker test is complex. Implementation of a neurocognitive blood test is expected to revolutionize the dementia diagnostic work‐up toward a more effective, more accessible, less expensive, less time‐consuming process with a lower burden for the patients in need of a timely and accurate, biological neurocognitive diagnosis.

## CONFLICT OF INTEREST STATEMENT

Jolien Jutte, Maurice Y. Kingma, Sinthujah Vigneswaran, Mariam M.T.E.E. Gouda, Marie‐Paule van Engelen, Claire Chevalier, Moira Marizzoni, Afina W. Lemstra, Yolande A.L. Pijnenburg, Anouk den Braber, and Martijn C. Schut have nothing to disclose. Inge M.W. Verberk received a speaker honorarium from Quanterix, which was paid directly to her institution. Daniel Alcolea received research grants from Pla Estratègic de Recerca i Innovació en Salut (PERIS SLT006/17/125), and from Instituto de Salud Carlos III (PI18/00435, PI22/00611, INT19/00016 and INT23/00048), participated in advisory boards from Fujirebio‐Europe, Roche Diagnostics, Grifols S.A. and Lilly, and received speaker honoraria from Fujirebio‐Europe, Roche Diagnostics, Nutricia, Krka Farmacéutica S.L., Zambon S.A.U. and Esteve Pharmaceuticals S.A. Daniel Alcolea declares a filed patent application (WO2019175379 A1 Markers of synaptopathy in neurodegenerative disease). Javier Arranz received funding from a “Rio Hortega” research grant from the Institute of Health Carlos III. Juan Fortea received research grants from Institute of Health Carlos III, National Institutes of Health, Fundació La Marató de TV3, and Pla Estratègic de Recerca i Innovació en Salut (PERIS). Juan Fortea has served as a consultant for Novartis and Lundbeck, has received honoraria for lectures from Roche, NovoNordisk, Esteve and Biogen and served at advisory boards for AC Immune, Zambon and Lundbeck. Juan Fortea declares a filed patent application (WO2019175379 A1 Markers of synaptopathy in neurodegenerative disease). Alberto Lleó received research grants from CIBERNED, Institute of Health Carlos III, Generalitat de Catalunya (PERIS and AGAUR) and Fundación Tatiana and BBVA. Alberto Lleó participated in advisory boards from Biogen, Eisai, Fujirebio‐Europe, Novartis, NovoNordisk, Nutricia, Otsuka Pharmaceutical, and Zambón, and received speaker honoraria from Lilly, Biogen, KRKA and Zambon. Alberto Lleó declares a filed patent application (WO2019175379 A1 Markers of synaptopathy in neurodegenerative disease). Elsmarieke M. van de Giessen has performed contract research for Heuron Inc. and Roche. Elsmarieke M. van de Giessen has a consultancy agreement with IXICO for the reading of PET scans. All funding is paid directly to her institution. Wiesje M. van der Flier has performed contract research for Biogen MA Inc, and Boehringer Ingelheim. Wiesje M. van der Flier has been an invited speaker at Boehringer Ingelheim, Biogen MA Inc, Danone, Eisai, WebMD Neurology (Medscape), Springer Healthcare. Wiesje M. van der Flier is consultant to Oxford Health Policy Forum CIC, Roche, and Biogen MA Inc. Wiesje M. van der Flier participated in advisory boards of Biogen MA Inc and Roche. All funding is paid to her institution. Wiesje M. van der Flier is a member of the steering committee of PAVE, and Think Brain Health. Wiesje M. van der Flier was associate editor of Alzheimer, Research & Therapy in 2020/2021, and is currently an associate editor at Brain. David Wilson is an employee of Quanterix. Argonde C. van Harten is a member of the advisory board of Brain Research Center. Charlotte E. Teunissen performed contract research for Acumen, ADx Neurosciences, AC‐Immune, Alamar, Aribio, Axon Neurosciences, Beckman‐Coulter, BioConnect, Bioorchestra, Brainstorm Therapeutics, Celgene, Cognition Therapeutics, EIP Pharma, Eisai, Eli Lilly, Fujirebio, Grifols, Instant Nano Biosensors, Merck, Novo Nordisk, Olink, PeopleBio, Quanterix, Roche, Toyama, Vivoryon. Charlotte E. Teunissen is editor in chief of Alzheimer Research and Therapy, and serves on editorial boards of Medidact Neurologie/Springer, and Neurology: Neuroimmunology & Neuroinflammation. Author disclosures are available in the Supporting information.

## CONSENT STATEMENT

All human subjects provided written informed consent for use of biomaterials and medical data for the scientific research purposes presented in this article.

## Supporting information

Supporting Information

Supporting Information
