# Supplementary material for: Development of thresholds and a visualization tool for use of a blood test in routine clinical dementia practice
Source: Alzheimers Dement. 2024 Aug 3;20(9):6115–32. doi: 10.1002/alz.14088 (PMC11497719; doi:10.1002/alz.14088)
Supplement: Supplementary file 1 — Supporting Information [file ALZ-20-6115-s002.docx]

**SUPPLEMENTARY MATERIAL**

**Development of thresholds and a visualization tool for use of a blood test in routine clinical dementia practice.**

Inge M.W. Verberk, PhD, Jolien Jutte MSc, Maurice Y. Kingma, MSc, Sinthujah Vigneswaran, MD, Mariam M.T.E.E. Gouda, MSc, Marie-Paule van Engelen, MD, Daniel Alcolea, MD PhD, Javier Arranz, MD, Juan Fortea, MD PhD, Alberto Lleó, MD PhD, Claire Chevalier, PhD, Moira Marizzoni, PhD, Elsmarieke van de Giessen, MD PhD, Afina W. Lemstra, MD PhD, Yolande A.L. Pijnenburg, MD PhD, Wiesje M. van der Flier, PhD, Anouk den Braber, PhD, David Wilson, PhD, Martijn C. Schut, PhD, Argonde C. van Harten, MD PhD1, Charlotte E. Teunissen, PhD

**Supplementary table 1. Inter-assay %CV of the Simoa measurements.**

|  | **Amsterdam Dementia Cohort** | **Geneva** | **Barcelona** |
| --- | --- | --- | --- |
| Abeta1-42 | 7% | 7% | 7% |
| Abeta1-40 | 8% | 4% | 3% |
| GFAP | 14% | 13% | 8% |
| NfL | 8% | 4% | 10% |
| P-tau181 | 8% | 12% | 8% |

*Inter-assay % coefficient of variation is presented of quality control (QC) samples included in each Simoa N4PE run (Amsterdam Dementia Cohort: 3 QCs in 6 runs; Geneva: 2 QCs in 3 runs; Barcelona: 3 QCs in 6 runs) and P-tau181 run (Amsterdam Dementia Cohort: 2 QCs in 12 runs; Geneva: 2 QCs in 4 runs; Barcelona: 2 QCs in 13 runs). Abeta = amyloid beta, GFAP = glial fibrillary acidic protein, NfL = neurofilament light, P-tau = phosphorylated tau.*

**
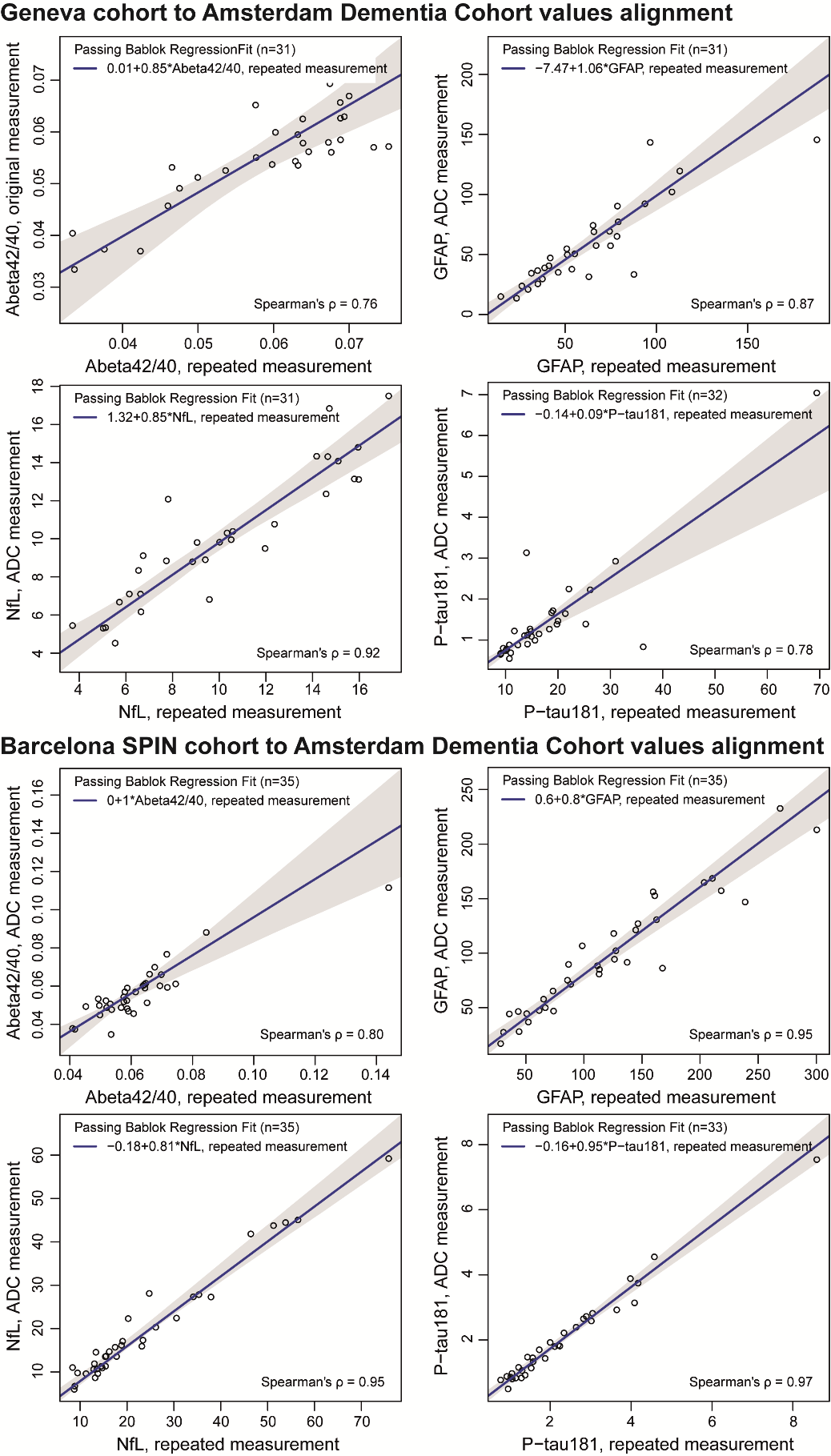
**

**Supplementary figure 1.** *Transformation formulas to align the validation cohort values (top panel: Geneva cohort; lower panel: Barcelona SPIN cohort) to the Amsterdam Dementia Cohort (ADC) values are presented. Formulas were calculated with Passing Bablok regression, based on 32 ADC samples for the Geneva cohort and 35 ADC samples for the Barcelona SPIN cohort that were re-measured with the respective Geneva and Barcelona SPIN cohort neurology 4-plex E and p-tau181 Simoa kit lot numbers. Abeta = amyloid beta, GFAP = glial fibrillary acidic protein, NfL = neurofilament light, P-tau = phosphorylated tau.*

**Supplementary table 2. Bonferroni-corrected p-values for all clinical characteristic and biomarker group comparisons within the Amsterdam Dementia Cohort.**

|  | **Demographics** | | | | | **CSF markers** | | | **Plasma markers** | | | |
| --- | --- | --- | --- | --- | --- | --- | --- | --- | --- | --- | --- | --- |
|  | ***Age*** | ***Sex*** | ***APOEε4*** | ***MMSE*** | ***Education*** | ***Abeta42*** | ***P-tau181*** | ***T-tau*** | ***Abeta42/40*** | ***P-tau181*** | ***GFAP*** | ***NfL*** |
| SCD Aβ- vs SCD Aβ+ | <0.001 | 1.000 | <0.001 | 1.000 | 1.000 | <0.001 | <0.001 | <0.001 | <0.001 | <0.001 | <0.001 | <0.001 |
| SCD Aβ- vs MCI Aβ- | <0.001 | 0.001 | 1.000 | <0.001 | 0.016 | 1.000 | 1.000 | 1.000 | *0.213 | 1.000 | 1.000 | 0.003 |
| SCD Aβ- vs MCI Aβ+ | <0.001 | 1.000 | <0.001 | <0.001 | 1.000 | <0.001 | <0.001 | <0.001 | <0.001 | <0.001 | <0.001 | <0.001 |
| SCD Aβ- vs AD dementia | <0.001 | 0.002 | <0.001 | <0.001 | 0.014 | <0.001 | <0.001 | <0.001 | <0.001 | <0.001 | <0.001 | <0.001 |
| SCD Aβ- vs FTD | <0.001 | 1.000 | 1.000 | <0.001 | <0.001 | 0.020 | *0.161 | <0.001 | <0.001 | *0.516 | <0.001 | <0.001 |
| SCD Aβ- vs DLB | <0.001 | <0.001 | <0.001 | <0.001 | 0.070 | <0.001 | 0.009 | <0.001 | <0.001 | <0.001 | <0.001 | <0.001 |
| SCD Aβ+ vs MCI Aβ- | 0.001 | 0.001 | <0.001 | 0.047 | 0.009 | <0.001 | <0.001 | <0.001 | <0.001 | <0.001 | <0.001 | 0.007 |
| SCD Aβ+ vs MCI Aβ+ | 1.000 | 1.000 | 1.000 | <0.001 | *0.387 | *0.334 | 1.000 | 1.000 | 1.000 | 1.000 | 1.000 | 1.000 |
| SCD Aβ+ vs AD dementia | 0.008 | 1.000 | 1.000 | <0.001 | 0.017 | 0.002 | 0.014 | <0.001 | 1.000 | *0.056 | *0.287 | 1.000 |
| SCD Aβ+ vs FTD | 0.003 | 1.000 | <0.001 | <0.001 | <0.001 | <0.001 | <0.001 | *0.146 | 0.009 | <0.001 | *0.509 | <0.001 |
| SCD Aβ+ vs DLB | 1.000 | <0.001 | *0.315 | <0.001 | 0.026 | 1.000 | <0.001 | 0.002 | 1.000 | *0.137 | 1.000 | 1.000 |
| MCI Aβ- vs MCI Aβ+ | 0.002 | 0.002 | <0.001 | 1.000 | 1.000 | <0.001 | <0.001 | <0.001 | <0.001 | <0.001 | <0.001 | 0.005 |
| MCI Aβ- vs AD dementia | 1.000 | <0.001 | <0.001 | <0.001 | 1.000 | <0.001 | <0.001 | <0.001 | <0.001 | <0.001 | <0.001 | <0.001 |
| MCI Aβ- vs FTD | 1.000 | <0.001 | 1.000 | <0.001 | 1.000 | *0.464 | 1.000 | <0.001 | 1.000 | 1.000 | <0.001 | <0.001 |
| MCI Aβ- vs DLB | <0.001 | 1.000 | 0.001 | <0.001 | 1.000 | <0.001 | *0.487 | *0.060 | 0.006 | 0.023 | <0.001 | <0.001 |
| MCI Aβ+ vs AD dementia | 0.010 | 0.047 | 0.020 | <0.001 | 1.000 | 1.000 | 0.041 | <0.001 | 1.000 | 0.005 | 0.004 | 0.046 |
| MCI Aβ+ vs FTD | 0.004 | 1.000 | <0.001 | <0.001 | 0.030 | <0.001 | <0.001 | <0.001 | <0.001 | <0.001 | *0.092 | <0.001 |
| MCI Aβ+ vs DLB | *0.449 | <0.001 | 0.004 | <0.001 | 1.000 | <0.001 | <0.001 | <0.001 | *0.774 | 0.002 | 1.000 | 1.000 |
| AD dementia vs FTD | 1.000 | *0.530 | <0.001 | <0.001 | *0.276 | <0.001 | <0.001 | <0.001 | <0.001 | <0.001 | <0.001 | <0.001 |
| AD dementia vs DLB | <0.001 | <0.001 | 1.000 | *0.210 | 1.000 | <0.001 | <0.001 | <0.001 | 1.000 | <0.001 | 0.001 | 1.000 |
| FTD vs DLB | <0.001 | <0.001 | <0.001 | *1.000 | 1.000 | <0.001 | 1.000 | 1.000 | *0.193 | *0.125 | 1.000 | <0.001 |

*Chi-squared tests with Bonferroni correction for multiple testing (Sex, APOEε4 carriage) or Kruskall-Wallis tests (all other variables) with post-hoc Dunn’s testing with Bonferroni correction were applied to compare all groups. SCD = subjective cognitive decline, MCI = mild cognitive impairment, AD = Alzheimer’s disease, FTD = frontotemporal dementia, DLB = dementia with Lewy bodies, APOE = apolipoprotein E, MMSE = mini-mental state examination, Abeta = amyloid beta, P-tau = phosphorylated tau, T-tau = total tau, GFAP = glial fibrillary acidic protein, NfL = neurofilament light.* *p<0.05 when no Bonferroni multiple testing correction is applied.

**Supplementary figure 2. Boxplots of plasma biomarkers according to diagnostic group.**


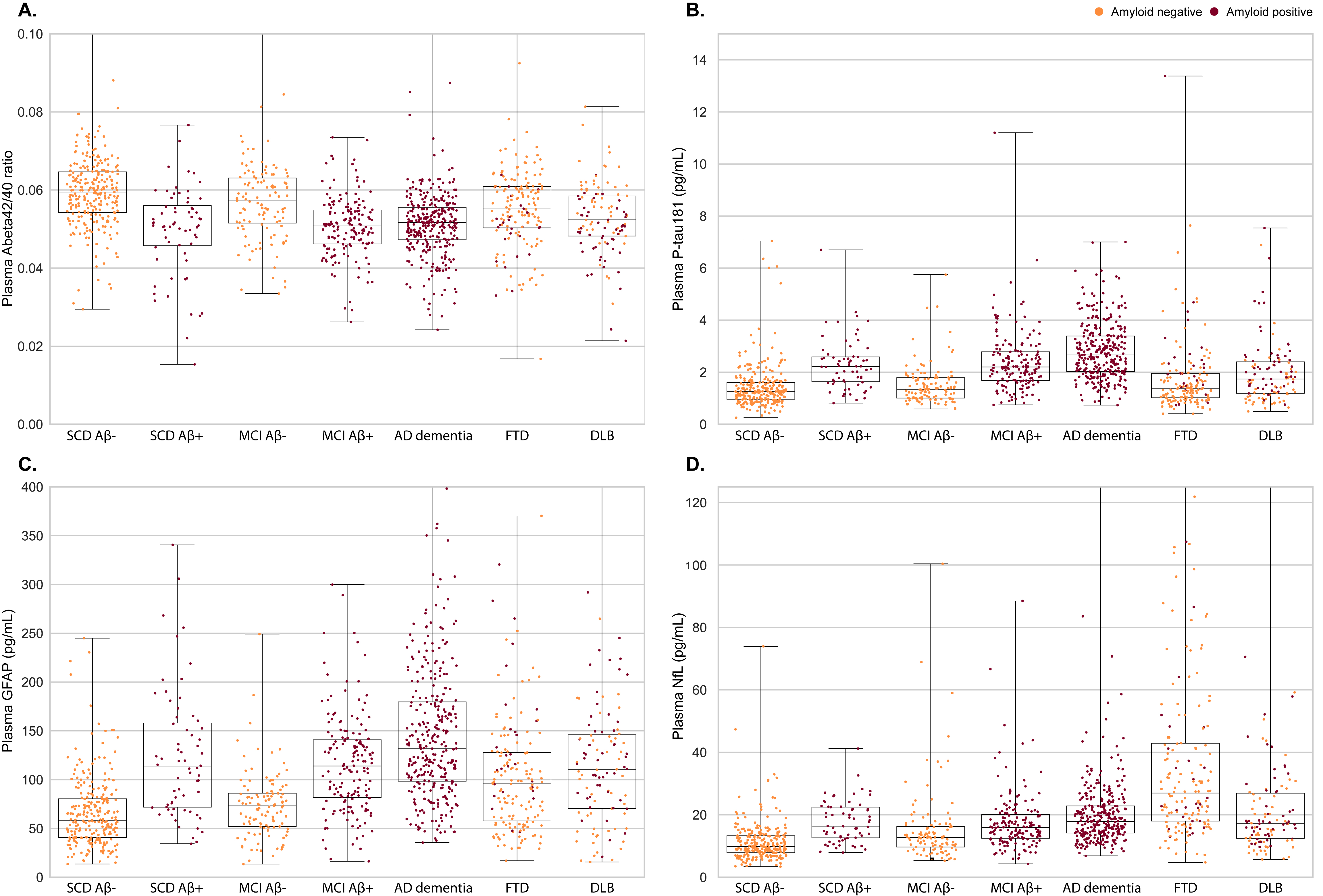


*Individuals with a negative amyloid status (Aβ-) are presented in orange, those with a positive amyloid status (Aβ+) are presented in red. Amyloid status was according to amyloid PET (n=309), cerebrospinal fluid P-tau181/Abeta42 ratio measured with Innotest (n=741), Elecsys (n=113) or a combination of Innotest and Elecsys (n=32), or taken from the patient files (n=4). SCD = subjective cognitive decline, MCI = mild cognitive impairment, AD = Alzheimer’s disease, FTD = frontotemporal dementia, DLB = dementia with Lewy bodies, Abeta = amyloid beta, P-tau = phosphorylated tau, GFAP = glial fibrillary acidic protein, NfL = neurofilament light.*

**Supplementary table 3. Youden’s index thresholds with sensitivity and specificity determined for the LASSO-selected biomarker for the six clinical questions.**

|  | **P-tau181** | | | **GFAP** | | | **Age-corrected NfL** | | |
| --- | --- | --- | --- | --- | --- | --- | --- | --- | --- |
|  | ***Threshold*** | ***Sens*** | ***Spec*** | ***Threshold*** | ***Sens*** | ***Spec*** | ***Threshold*** | ***Sens*** | ***Spec*** |
| 1. Aβ+/Aβ- in total cohort | 1.81 | 0.76 | 0.80 | 102 | 0.67 | 0.78 | -0.17 | 0.80 | 0.41 |
| 2. Aβ+/Aβ- in SCD + MCI | 1.64 | 0.78 | 0.75 | 87.2 | 0.68 | 0.79 | -0.17 | 0.73 | 0.54 |
| 3. AD vs FTD | 1.71 | 0.87 | 0.70 | 111 | 0.67 | 0.65 | 1.79 | 0.92 | 0.43 |
| 4. Controls vs FTD | 1.28 | 0.59 | 0.54 | 87.3 | 0.58 | 0.80 | 0.56 | 0.79 | 0.87 |
| 5. AD vs DLB | 1.81 | 0.84 | 0.56 | 111 | 0.67 | 0.51 | 0.22 | 0.70 | 0.54 |
| 6. Controls vs DLB | 1.70 | 0.54 | 0.81 | 102 | 0.60 | 0.88 | -0.16 | 0.69 | 0.57 |

*Thresholds at Youden***’***s indices were computed based on ROC analysis over the total data subsets, without internal cross-validation. Thresholds are in pg/mL for P-tau181 and GFAP, whereas they are age-corrected Z-scores for NfL. NfL was corrected for age using a published formula.^46^ Please note that these thresholds are Simoa kit lot dependent, and a value alignment factor is needed to apply the thresholds to new data obtained with other kit lots. Aβ = amyloid status, SCD = subjective cognitive decline, MCI = mild cognitive impairment, AD = Alzheimer’s disease, FTD = frontotemporal dementia, DLB = dementia with Lewy bodies, P-tau = phosphorylated tau, GFAP = glial fibrillary acidic protein, NfL = neurofilament light, sens = sensitivity, spec = specificity.*

**Supplementary table 4. Logistic regression probability thresholds at Youden’s index, at 90% specificity and 90% sensitivity for P-tau181, GFAP and age-corrected NfL interpreted in aggregation for the six clinical questions.**

|  | **Youden’s index** | | | **90% Specificity** | | | **90% Sensitivity** | | |
| --- | --- | --- | --- | --- | --- | --- | --- | --- | --- |
|  | ***Threshold*** | ***Sens*** | ***Spec*** | ***Threshold*** | ***Sens*** | ***Spec*** | ***Threshold*** | ***Sens*** | ***Spec*** |
| 1. Aβ+/Aβ- in total cohort | 0.522 | 0.83 | 0.75 | 0.345 | 0.900 | 0.58 | 0.668 | 0.900 | 0.61 |
| 2. Aβ+/Aβ- in SCD + MCI | 0.399 | 0.85 | 0.70 | 0.219 | 0.900 | 0.58 | 0.519 | 0.900 | 0.52 |
| 3. AD vs FTD | 0.512 | 0.71 | 0.96 | 0.608 | 0.900 | 0.53 | 0.850 | 0.900 | 0.76 |
| 4. Controls vs FTD | 0.423 | 0.89 | 0.79 | 0.202 | 0.900 | 0.76 | 0.457 | 0.900 | 0.66 |
| 5. AD vs DLB | 0.662 | 0.57 | 0.82 | 0.608 | 0.900 | 0.23 | 0.876 | 0.900 | 0.44 |
| 6. Controls vs DLB | 0.375 | 0.88 | 0.62 | 0.136 | 0.900 | 0.54 | 0.417 | 0.900 | 0.26 |

*Patient’ probabilities of the logistic regression including the LASSO-selcted biomarkers P-tau181, GFAP and age-corrected NfL were calculated for each clinical question, and used in ROC analysis over the total data subsets without internal cross-validation to compute thresholds with their corresponding specificities and sensitivities at Youden’s index and at 90% specificity and 90% sensitivity. NfL was corrected for age using a published formula.^46^ Aβ = amyloid status, SCD = subjective cognitive decline, MCI = mild cognitive impairment, AD = Alzheimer’s disease, FTD = frontotemporal dementia, DLB = dementia with Lewy bodies, P-tau = phosphorylated tau, GFAP = glial fibrillary acidic protein, NfL = neurofilament light, sens = sensitivity, spec = specificity.*

**Supplementary table 5. Linear predictor logistic regression formulas to calculate probability scores for the LASSO-selected biomarkers P-tau181, GFAP and age-corrected NfL.**

|  | **Linear predictor logistic regression formula** |
| --- | --- |
| 1. Aβ+/Aβ- in total cohort | 1/(1+e^(-(-3.2901 + 0.0171 * GFAP + 0.9536 * pTau181 + -0.4170 * age-corrected NfL))) |
| 2. Aβ+/Aβ- in SCD + MCI | 1/(1+e^(-(-3.6418 + 0.0216 * GFAP + 0.6826 * pTau181 + -0.0974 * age-corrected NfL))) |
| 3. AD vs FTD | 1/(1+e^(-(-1.5706 + 0.0121 * GFAP + 0.9003 * pTau181 + -1.2229 * age-corrected NfL))) |
| 4. Controls vs FTD | 1/(1+e^(-(-1.9429 + 0.0100 * GFAP + -0.1182 * pTau181 + 1.6148 * age-corrected NfL))) |
| 5. AD vs DLB | 1/(1+e^(-(-0.7347 + -0.0008 * GFAP + 0.8100 * pTau181 + 0.0047 * age-corrected NfL))) |
| 6. Controls vs DLB | 1/(1+e^(-(-2.6705 + 0.0198 * GFAP + 0.0492 * pTau181 + 0.2225 * age-corrected NfL))) |

*Logistic regression including the LASSO-selected biomarkers P-tau181, GFAP and age-corrected NfL on the total data subsets without internal cross validation was applied for each clinical question, resulting in the linear predictor formulas presented here. Biomarker levels are in pg/mL for P-tau181 and GFAP, whereas they are age-corrected Z-scores for NfL. NfL was corrected for age using a published formula.^46^ Please note that the biomarker levels are Simoa kit lot dependent, and a value alignment factor is needed to apply the thresholds to new data obtained with other kit lots. Aβ = amyloid status, SCD = subjective cognitive decline, MCI = mild cognitive impairment, AD = Alzheimer’s disease, FTD = frontotemporal dementia, DLB = dementia with Lewy bodies, P-tau = phosphorylated tau, GFAP = glial fibrillary acidic protein, NfL = neurofilament light.*

**Supplementary table 6. Proportions of individuals that had probabilities falling in the low, intermediate or high likelihood groups of the density plots for each clinical question, including the percentages of individuals that had a particular diagnosis for each likelihood group.**

|  | **Low likelihood** | **Intermediate/undetermined likelihood** | **High likelihood** |
| --- | --- | --- | --- |
| **1. Aβ+/- in total cohort** | **34%** | **30%** | **35%** |
| *Aβ-* | *84%* | *33%* | *13%* |
| *Aβ+* | *16%* | *26%* | *87%* |
| **2. Aβ+/- in SCD + MCI** | **36%** | **35%** | **28%** |
| *Aβ-* | *89%* | *57%* | *21%* |
| *Aβ+* | *11%* | *21%* | *79%* |
| **3. FTD vs AD** | **31%** | **33%** | **36%** |
| *FTD* | *78%* | *4%* | *8%* |
| *AD* | *22%* | *10%* | *92%* |
| **4. Controls vs FTD** | **45%** | **20%** | **35%** |
| *Control* | *91%* | *69%* | *16%* |
| *FTD* | *9%* | *20%* | *84%* |
| **5. DLB vs AD** | **19%** | **62%** | **20%** |
| *DLB* | *59%* | *6%* | *12%* |
| *AD* | *41%* | *10%* | *88%* |
| **6. Controls vs DLB** | **22%** | **56%** | **23%** |
| *Control* | *85%* | *78%* | *29%* |
| *DLB* | *15%* | *15%* | *71%* |

*Probabilities of logistic regression analysis combining P-tau181, GFAP and age-corrected NfL were calculated for each clinical question and interpreted according to probability threshold lines which were set at 90% specificity and at 90% sensitivity for each clinical question. The low likelihood group contains individuals that score a probability ranging from 0 to the 90% specificity probability thresholds. The intermediate/undetermined likelihood group contains individuals that score a probability ranging from the 90% specificity probability thresholds to the 90% sensitivity thresholds. The high likelihood group contains individuals that score a probability ranging from the 90% sensitivity probability threshold until 1. In bold and underlined, the % of individuals falling in each group (low/intermediate/high) is presented for each question, and in italic the % per group per question scoring a certain diagnosis are presented. NfL was corrected for age using a published formula.^46^ Aβ = amyloid status, SCD = subjective cognitive decline, MCI = mild cognitive impairment, FTD = frontotemporal dementia, AD = Alzheimer’s disease, DLB = dementia with Lewy bodies.*

**Supplementary table 7. Demographics and clinical characteristics of the Geneva Cohort.**

|  | **CN Aβ-** | **CN Aβ+** | **MCI Aβ-** | **MCI Aβ+** | **Dementia Aβ-** | **Dementia Aβ+** |
| --- | --- | --- | --- | --- | --- | --- |
|  | *n=84* | *n=21* | *n=56* | *n=64* | *n=9* | *n=35* |
| **Demographics** |  |  |  |  |  |  |
| Age, years | 66 ± 8 | 66 ± 9 | 68 ± 9 | 74 ± 6 | 73 ± 8 | 75 ± 5 |
| Female sex | 51 (61) | 12 (57) | 24 (43) | 33 (52) | 5 (56) | 16 (46) |
| APOEε4 carriage | 7 (8) | 6 (29) | 7 (13) | 30 (47) | 0 (0) | 10 (29) |
| MMSE | 29 ± 1.4 | 29 ± 1.6 | 27 ± 2.2 | 26 ± 2.5 | 21 ± 3.3 | 19 ± 5.2 |
| Education, years | 15 ± 3.9 | 15 ± 3.0 | 13 ± 4.0 | 13 ± 3.9 | 9 ± 5.7 | 12 ± 4.0 |
|  |  |  |  |  |  |  |
| **CSF markers, Innotest** | |  |  |  |  |  |
| Abeta42, pg/mL | 1180 ± 295 | 715 ± 141 | 1214 ± 441 | 562 ± 146 | 1268 ± 325 | 486 ± 147 |
| P-tau181, pg/mL | 41 ± 16 | 53 ± 53 | 56 ± 20 | 81 ± 34 | 39 ± 7 | 92 ± 57 |
| T-tau, pg/mL | 268 ± 105 | 353 ± 297 | 308 ± 172 | 531 ± 264 | 299 ± 102 | 631 ± 556 |
| **CSF markers, Lumipulse** | |  |  |  |  |  |
| Abeta42/40 ratio | 0.32 ± 0.46 | 0.05 ± 0.00 | 0.11 ± 0.01 | 0.05 ± 0.01 | 0.10 ± 0.02 | 0.05 ± 0.01 |
| P-tau181, pg/mL | 40 ± 15 | 94 ± 14 | 44 ± 22 | 102 ± 41 | 50 ± 12 | 122 ± 50 |
| T-tau, pg/mL | 303 ± 125 | 625 ± 57 | 336 ± 158 | 649 ± 239 | 443 ± 169 | 750 ± 316 |
|  |  |  |  |  |  |  |
| **Plasma markers** |  |  |  |  |  |  |
| Abeta42/40 | 0.07 ± 0.02 | 0.06 ± 0.01 | 0.07 ± 0.01 | 0.06 ± 0.01 | 0.07 ± 0.01 | 0.06 ± 0.01 |
| P-tau181, pg/mL | 1.79 ± 0.69 | 2.22 ± 0.74 | 1.85 ± 1.20 | 2.77 ± 0.97 | 1.54 ± 0.39 | 3.70 ± 1.37 |
| GFAP, pg/mL | 112 ± 57 | 156 ± 79 | 150 ± 240 | 203 ± 89 | 138 ± 54 | 284 ± 125 |
| NfL, pg/mL | 16.2 ± 6.6 | 17.5 ± 7.7 | 18.1 ± 10 | 25.8 ± 24.9 | 27.9 ± 25 | 30.7 ± 10 |

*Data is presented as mean ± SD or n (%), for only the 269 cases with a diagnosis within one year of the plasma results. Amyloid status was according to amyloid PET (n=127) or based on CSF measurement (n=142). CSF biomarkers were measured with either Innotest (n=75) or Lumipulse (n=67). APOE ε4 carriage is defined when an individual carries at least one APOE ε4 allele. MMSE was missing for n=4, APOE* ε4 carriage *was missing for n=121, CSF biomarkers were missing for n=116. CN = cognitively normal, MCI = mild cognitive impairment, APOE = apolipoprotein E, MMSE = mini-mental state examination, Abeta = amyloid beta, P-tau = phosphorylated tau, T-tau = total tau, GFAP = glial fibrillary acidic protein, NfL = neurofilament light. Note: for the comparisons Aβ- vs. Aβ+ in the results section, all 313 participants were included.*

**Supplementary table 8. Bonferroni-corrected p-values for all clinical characteristic and biomarker group comparisons within the Geneva cohort.**

|  | **Demographics** | | | | | **CSF markers** | | | **Plasma markers** | | | |
| --- | --- | --- | --- | --- | --- | --- | --- | --- | --- | --- | --- | --- |
|  | ***Age*** | ***Sex*** | ***APOEε4*** | ***MMSE*** | ***Education*** | ***Abeta42*** | ***P-tau181*** | ***T-tau*** | ***Abeta42/40*** | ***P-tau181*** | ***GFAP*** | ***NfL*** |
| CN Aβ- vs CN Aβ+ | 1.000 | 1.000 | 1.000 | 1.000 | 1.000 | *0.053 | 1.000 | 1.000 | 0.001 | *0.293 | *0.144 | 1.000 |
| CN Aβ- vs MCI Aβ- | 0.826 | 1.000 | 1.000 | 0.002 | 0.006 | 1.000 | 1.000 | 1.000 | 1.000 | 1.000 | 1.000 | 1.000 |
| CN Aβ- vs MCI Aβ+ | <0.001 | 1.000 | *0.060 | <0.001 | *0.065 | <0.001 | <0.001 | <0.001 | <0.001 | <0.001 | <0.001 | <0.001 |
| CN Aβ- vs dementia Aβ- | *0.156 | 1.000 | 1.000 | <0.001 | 0.004 | 1.000 | 1.000 | 1.000 | 1.000 | 1.000 | 1.000 | 0.792 |
| CN Aβ- vs dementia Aβ+ | <0.001 | 1.000 | 1.000 | <0.001 | 0.001 | <0.001 | <0.001 | <0.001 | <0.001 | <0.001 | <0.001 | <0.001 |
| CN Aβ+ vs MCI Aβ- | 1.000 | 1.000 | 1.000 | *0.147 | *0.381 | 0.001 | 1.000 | 1.000 | 0.001 | *0.207 | *0.717 | 1.000 |
| CN Aβ+ vs MCI Aβ+ | 0.011 | 1.000 | 1.000 | <0.001 | 1.000 | 1.000 | 0.025 | *0.056 | 1.000 | 0.813 | 1.000 | *0.319 |
| CN Aβ+ vs dementia Aβ- | 0.787 | 1.000 | 1.000 | <0.001 | 0.028 | *0.139 | 1.000 | 1.000 | 0.854 | *0.538 | 1.000 | 1.000 |
| CN Aβ+ vs dementia Aβ+ | 0.002 | 1.000 | 1.000 | <0.001 | *0.063 | *0.522 | 0.001 | 0.007 | 1.000 | 0.004 | 0.007 | <0.001 |
| MCI Aβ- vs MCI Aβ+ | 0.007 | 1.000 | 0.002 | *0.123 | 1.000 | <0.001 | <0.001 | <0.001 | <0.001 | <0.001 | <0.001 | 0.016 |
| MCI Aβ- vs dementia Aβ- | 1.000 | 1.000 | 1.000 | 0.002 | 0.945 | 1.000 | 1.000 | 1.000 | 1.000 | 1.000 | 1.000 | 1.000 |
| MCI Aβ- vs dementia Aβ+ | 0.001 | 1.000 | 0.910 | <0.001 | 1.000 | <0.001 | <0.001 | <0.001 | <0.001 | <0.001 | <0.001 | <0.001 |
| MCI Aβ+ vs dementia Aβ- | 1.000 | 1.000 | *0.069 | *0.192 | *0.358 | <0.001 | *0.087 | 1.000 | *0.345 | 0.003 | 1.000 | 1.000 |
| MCI Aβ+ vs dementia Aβ+ | 1.000 | 1.000 | 1.000 | <0.001 | 1.000 | 1.000 | 1.000 | 1.000 | 1.000 | *0.214 | *0.174 | 0.018 |
| dementia Aβ- vs dementia Aβ+ | 1.000 | 1.000 | 1.000 | 1.000 | 1.000 | <0.001 | 0.009 | *0.583 | 1.000 | <0.001 | 0.026 | *0.667 |

*Chi-squared tests with Bonferroni correction for multiple testing (Sex, APOEε4 carriage) or Kruskall-Wallis tests (all other variables) with post-hoc Dunn’s testing with Bonferroni correction were applied to compare all groups. The Innotest and Lumipulse CSF markers were first Z-scored and combined prior to group comparison analysis. CN = cognitively normal, MCI = mild cognitive impairment, Aβ = amyloid status, APOE = apolipoprotein E, MMSE = mini-mental state examination, Abeta = amyloid beta, P-tau = phosphorylated tau, T-tau = total tau, GFAP = glial fibrillary acidic protein, NfL = neurofilament light.* *p<0.05 when no Bonferroni multiple testing correction is applied.

**Supplementary table 9. Demographics and clinical characteristics of the Barcelona Cohort.**

|  | **CN Aβ-** | **CN Aβ+** | **MCI Aβ-** | **MCI Aβ+** | **AD dementia** | **FTD** | **DLB** |
| --- | --- | --- | --- | --- | --- | --- | --- |
|  | *n=20* | *n=11* | *n=37* | *n=25* | *n=23* | *n=56* | *n=68* |
| **Demographics** |  |  |  |  |  |  |  |
| Age, years | 63 ± 5 | 73 ± 9 | 71 ± 6 | 73 ± 5 | 71 ± 7 | 72 ± 9 | 76 ± 5 |
| Female sex | 11 (55) | 5 (45) | 18 (49) | 15 (60) | 14 (61) | 24 (43) | 30 (44) |
| APOEε4 carriage | 1 (5) | 3 (27) | 9 (24) | 9 (36) | 12 (52) | 9 (16) | 22 (32) |
| MMSE | 29 ± 0.9 | 28 ± 1.5 | 26 ± 2.3 | 26 ± 1.7 | 21 ± 4.7 | 22 ± 4.8 | 22 ± 4.5 |
| Education, years | 16 ± 3.6 | 13 ± 4.9 | 10 ± 4.4 | 11 ± 4.9 | 12 ± 5.5 | 11 ± 5.3 | 9.2 ± 4.8 |
|  |  |  |  |  |  |  |  |
| **CSF markers** |  |  |  |  |  |  |  |
| Abeta42/40 ratio | 0,.11 ± 0.01 | 0.05 ± 0.01 | 0.10 ± 0.02 | 0.04 ± 0.01 | 0.05 ± 0.01 | 0.09 ± 0.01 | 0.07 ± 0.03 |
| P-tau181, pg/mL | 38 ± 10 | 87 ± 31 | 41 ± 13 | 132 ± 70 | 137 ± 79 | 42 ± 20 | 85 ± 70 |
| T-tau, pg/mL | 267 ± 70 | 541 ± 214 | 311 ± 176 | 812 ± 463 | 836 ± 417 | 349 ± 178 | 531 ± 416 |
|  |  |  |  |  |  |  |  |
| **Plasma markers** |  |  |  |  |  |  |  |
| Abeta42/40 | 0.07 ± 0.01 | 0.06 ± 0.01 | 0.06 ± 0.01 | 0.05 ± 0.01 | 0.06 ± 0.01 | 0.06 ± 0.01 | 0.06 ± 0.01 |
| P-tau181, pg/mL | 1.37 ± 0.65 | 2.18 ± 0.98 | 1.51 ± 1.00 | 2.64 ± 1.35 | 3.87 ± 1.69 | 1.75 ± 1.03 | 3.20 ± 1.84 |
| GFAP, pg/mL | 74.0 ± 25 | 105 ± 40 | 87.9 ± 40 | 146 ± 75 | 169 ± 85 | 115 ± 77 | 148 ± 67 |
| NfL, pg/mL | 10.5 ± 4.8 | 13.7 ± 4.0 | 16.0 ± 8.3 | 17.7 ± 8.5 | 26.2 ± 22 | 31.8 ± 22 | 28.8 ± 34 |

*Data is presented as mean ± SD or n (%). APOE* ε4 carriage is defined when an individual carries at least one APOE ε4 allele. *Amyloid status was according to cerebrospinal fluid Abeta42/40 ratio, measured with Lumipulse G600II. APOE* ε4 carriage *was missing for n=2, MMSE was missing for n=6, education was missing for n=6, CSF Abeta42/40 was missing for n=13 (n=1 FTD, n=12 DLB), CSF P-tau181 was missing for n=13, CSF T-tau was missing for n=12, plasma P-tau181 was missing for n=1. CN = cognitively normal, MCI = mild cognitive impairment, AD = Alzheimer’s disease, FTD = frontotemporal dementia, DLB = dementia with Lewy bodies, APOE = apolipoprotein E, MMSE = mini-mental state examination, Abeta = amyloid beta, P-tau = phosphorylated tau, T-tau = total tau, GFAP = glial fibrillary acidic protein, NfL = neurofilament light.*

**Supplementary table 10. Bonferroni-corrected p-values for all clinical characteristic and biomarker group comparisons within the Barcelona cohort.**

|  | **Demographics** | | | | | **CSF markers** | | | **Plasma markers** | | | |
| --- | --- | --- | --- | --- | --- | --- | --- | --- | --- | --- | --- | --- |
|  | ***Age*** | ***Sex*** | ***APOEε4*** | ***MMSE*** | ***Education*** | ***Abeta42/40*** | ***P-tau181*** | ***T-tau*** | ***Abeta42/40*** | ***P-tau181*** | ***GFAP*** | ***NfL*** |
| CN Aβ- vs CN Aβ+ | 0.005 | 1.000 | 1.000 | 1.000 | 1.000 | <0.001 | 0.009 | 0.030 | 0.009 | *0.569 | 1.000 | 1.000 |
| CN Aβ- vs MCI Aβ- | 0.021 | 1.000 | 1.000 | 0.029 | <0.001 | 1.000 | 1.000 | 1.000 | *0.398 | 1.000 | 1.000 | *0.352 |
| CN Aβ- vs MCI Aβ+ | <0.001 | 1.000 | *0.706 | 0.047 | 0.010 | <0.001 | <0.001 | <0.001 | <0.001 | 0.003 | 0.003 | *0.083 |
| CN Aβ- vs AD dementia | 0.025 | 1.000 | *0.052 | <0.001 | *0.370 | <0.001 | <0.001 | <0.001 | <0.001 | <0.001 | <0.001 | 0.002 |
| CN Aβ- vs FTD | <0.001 | 1.000 | 1.000 | <0.001 | 0.007 | 0.931 | 1.000 | 1.000 | 0.044 | 1.000 | *0.383 | <0.001 |
| CN Aβ- vs DLB | <0.001 | 1.000 | *0.650 | <0.001 | <0.001 | <0.001 | 0.017 | 0.033 | 0.001 | <0.001 | <0.001 | <0.001 |
| CN Aβ+ vs MCI Aβ- | 1.000 | 1.000 | 1.000 | 1.000 | *0.841 | <0.001 | 0.010 | *0.060 | 1.000 | *0.779 | 1.000 | 1.000 |
| CN Aβ+ vs MCI Aβ+ | 1.000 | 1.000 | 1.000 | 1.000 | 1.000 | 1.000 | 1.000 | 1.000 | 1.000 | 1.000 | 1.000 | 1.000 |
| CN Aβ+ vs AD dementia | 1.000 | 1.000 | 1.000 | <0.001 | 1.000 | 1.000 | 1.000 | 1.000 | 1.000 | *0.268 | *0.515 | *0.784 |
| CN Aβ+ vs FTD | 1.000 | 1.000 | 1.000 | 0.001 | 1.000 | <0.001 | 0.005 | *0.278 | 1.000 | 1.000 | 1.000 | 0.005 |
| CN Aβ+ vs DLB | 1.000 | 1.000 | 1.000 | <0.001 | *0.245 | *0.546 | 1.000 | 1.000 | 1.000 | 1.000 | 1.000 | 0.022 |
| MCI Aβ- vs MCI Aβ+ | 1.000 | 1.000 | 1.000 | 1.000 | 1.000 | <0.001 | <0.001 | <0.001 | 0.014 | 0.002 | 0.019 | 1.000 |
| MCI Aβ- vs AD dementia | 1.000 | 1.000 | 1.000 | <0.001 | 1.000 | <0.001 | <0.001 | <0.001 | *0.076 | <0.001 | <0.001 | 1.000 |
| MCI Aβ- vs FTD | 1.000 | 1.000 | 1.000 | <0.001 | 1.000 | 1.000 | 1.000 | 1.000 | 1.000 | 1.000 | 1.000 | <0.001 |
| MCI Aβ- vs DLB | 0.001 | 1.000 | 1.000 | <0.001 | 1.000 | <0.001 | 0.008 | 0.045 | 1.000 | <0.001 | <0.001 | 0.001 |
| MCI Aβ+ vs AD dementia | 1.000 | 1.000 | 1.000 | 0.003 | 1.000 | 1.000 | 1.000 | 1.000 | 1.000 | *0.773 | 1.000 | 1.000 |
| MCI Aβ+ vs FTD | 1.000 | 1.000 | 1.000 | 0.005 | 1.000 | <0.001 | <0.001 | <0.001 | *0.050 | 0.030 | *0.601 | 0.032 |
| MCI Aβ+ vs DLB | 1.000 | 1.000 | 1.000 | 0.001 | 1.000 | 0.001 | 0.005 | 0.007 | *0.802 | 1.000 | 1.000 | *0.184 |
| AD dementia vs FTD | 1.000 | 1.000 | *0.053 | 1.000 | 1.000 | <0.001 | <0.001 | <0.001 | *0.249 | <0.001 | 0.013 | 1.000 |
| AD dementia vs DLB | 0.041 | 1.000 | 1.000 | 1.000 | *0.289 | 0.015 | 0.004 | 0.002 | 1.000 | 1.000 | 1.000 | 1.000 |
| FTD vs DLB | *0.404 | 1.000 | 1.000 | 1.000 | *0.326 | 0.001 | 0.001 | *0.400 | 1.000 | <0.001 | 0.007 | 1.000 |

*Chi-squared tests with Bonferroni correction for multiple testing (Sex, APOEε4 carriage) or Kruskal-Wallis tests (all other variables) with post-hoc Dunn’s testing with Bonferroni correction were applied to compare all groups. CN = cognitively normal, MCI = mild cognitive impairment, AD = Alzheimer’s disease, FTD = frontotemporal dementia, DLB = dementia with Lewy bodies, APOE = apolipoprotein E, MMSE = mini-mental state examination, Abeta = amyloid beta, P-tau = phosphorylated tau, T-tau = total tau, GFAP = glial fibrillary acidic protein, NfL = neurofilament light.* *p<0.05 when no Bonferroni multiple testing correction is applied.

**Supplementary table 11. ROC-AUCs computed for relatedness of the Amsterdam Dementia Cohort characteristics and the validation cohorts Geneva and Barcelona characteristics**

|  | **Geneva** | **Barcelona** |
| --- | --- | --- |
| 1. Aβ+/Aβ- in total cohort | 0.759 | 0.652 |
| 2. Aβ+/Aβ- in CN/SCD + MCI | 0.777 | 0.690 |
| 3. AD vs FTD | / | 0.760 |
| 4. Controls vs FTD | / | 0.801 |
| 5. AD vs DLB | / | 0.726 |
| 6. Controls vs DLB | / | 0.784 |

*ROC-AUCs with 10-fold internal cross-validation from binary logistic membership models were computed for each clinical question including the LASSO-selected plasma markers P-tau181, GFAP and age-corrected NfL and the outcome measures diagnosis or amyloid status, dependent on the clinical question at hand. High AUCs are indicative of a different case mix, i.e., characteristics compare poorly.*

**Supplementary table 12. External validation results of the logistic regression models incorporating P-tau181, GFAP and age-corrected NfL developed in the Amsterdam Dementia Cohort.**

|  | **AUC-score validation cohort** | **AUC-score Amsterdam Dementia Cohort** | **Calibration- in-the-large** | **Calibration slope in logit space** |
| --- | --- | --- | --- | --- |
| **Geneva** |  |  |  |  |
| 1. Aβ+/Aβ- in total cohort | 0.84 [0.796 – 0.885] | 0.85 [0.828 – 0.872] | 0.242 | 0.103 |
| 2. Aβ+/Aβ- in CN + MCI | 0.82 [0.746 – 0.880] | 0.83 [0.790 – 0.861] | 0.210 | 0.091 |
| **Barcelona** |  |  |  |  |
| 1. Aβ+/Aβ- in total cohort | 0.83 [0.774 – 0.877] | 0.85 [0.828 – 0.872] | 0.228 | 0.103 |
| 2. Aβ+/Aβ- in CN + MCI | 0.78 [0.661 – 0.870] | 0.83 [0.790 – 0.861] | -0.012 | 0.121 |
| 3. AD vs FTD | 0.92 [0.844 – 0.976] | 0.87 [0.829 – 0.910] | 0.383 | 0.110 |
| 4. Controls vs FTD | 0.81 [0.702 – 0.900] | 0.90 [0.862 – 0.930] | -0.315 | 0.075 |
| 5. AD vs DLB | 0.63 [0.510 – 0.760] | 0.75 [0.690 – 0.799] | 0.546 | 0.048 |
| 6. Controls vs DLB | 0.88 [0.790 – 0.941] | 0.76 [0.704 – 0.827] | -0.280 | 0.126 |

*The logistic regression models including the LASSO-selected biomarkers P-tau181, GFAP and age-corrected NfL developed in the Amsterdam Dementia Cohort for the six clinical questions were applied to the external validation cohorts Geneva and Barcelona for the same clinical questions, to assess the models’ discrimination and calibration. Aβ = amyloid status, CN = cognitively normal, MCI = mild cognitive impairment, FTD = frontotemporal dementia, AD = Alzheimer’s disease, DLB = dementia with Lewy bodies, AUC = area under the curve.*

**Supplementary table 13. Accuracy, sensitivity and specificity metrics at Youden’s indices comparison between the Amsterdam Dementia Cohort and validation cohorts, when applying the Amsterdam Dementia Cohort models incorporating P-tau181, GFAP and age-corrected NfL to the external validation cohorts.**

|  | **Accuracy  validation cohort** | **Accuracy Amsterdam Dementia Cohort** | **Sensitivity  validation cohort** | **Sensitivity Amsterdam Dementia Cohort** | **Specificity  validation cohort** | **Specificity Amsterdam Dementia Cohort** |
| --- | --- | --- | --- | --- | --- | --- |
| **Geneva** |  |  |  |  |  |  |
| 1. Aβ+/Aβ- in total cohort | 0.689 | 0.784 | 0.921 | 0.749 | 0.503 | 0.829 |
| 2. Aβ+/Aβ- in CN + MCI | 0.650 | 0.783 | 0.958 | 0.704 | 0.413 | 0.849 |
| **Barcelona** |  |  |  |  |  |  |
| 1. Aβ+/Aβ- in total cohort | 0.695 | 0.784 | 0.860 | 0.749 | 0.829 | 0.593 |
| 2. Aβ+/Aβ- in CN + MCI | 0.685 | 0.783 | 0.694 | 0.704 | 0.849 | 0.676 |
| 3. AD vs FTD | 0.557 | 0.869 | 1.000 | 0.959 | 0.713 | 0.375 |
| 4. Controls vs FTD | 0.667 | 0.829 | 0.589 | 0.794 | 0.886 | 0.895 |
| 5. AD vs DLB | 0.440 | 0.778 | 0.870 | 0.822 | 0.574 | 0.294 |
| 6. Controls vs DLB | 0.736 | 0.790 | 0.721 | 0.620 | 0.882 | 0.789 |

*The logistic regression models including the LASSO-selected biomarkers P-tau181, GFAP and age-corrected NfL developed in the Amsterdam Dementia Cohort for the six clinical questions were applied to the external validation cohorts Geneva and Barcelona for the same clinical questions, to compare the agreement in accuracy, sensitivity and specificity at Youden’s indices. Aβ = amyloid status, CN = cognitively normal, MCI = mild cognitive impairment, FTD = frontotemporal dementia, AD = Alzheimer’s disease, DLB = dementia with Lewy bodies, AUC = area under the curve.*

**Supplementary table 14. Confusion matrix at Youden’s indices comparison between the Amsterdam Dementia Cohort and validation cohorts, when applying the Amsterdam Dementia Cohort models incorporating P-tau181, GFAP and age-corrected NfL to the external validation cohorts.**

|  | **TP  validation cohort** | **TP Amsterdam Dementia Cohort** | **TN  validation cohort** | **TN**  **Amsterdam Dementia Cohort** | **FP  validation cohort** | **FP**  **Amsterdam Dementia Cohort** | **FN  validation cohort** | **FN**  **Amsterdam Dementia Cohort** |
| --- | --- | --- | --- | --- | --- | --- | --- | --- |
| **Geneva** |  |  |  |  |  |  |  |  |
| 1. Aβ+/Aβ- in total cohort | 128 (41.03%) | 469 (39.64%) | 87 (27.88%) | 462 (39.05%) | 86 (27.56%) | 95 (8.03%) | 11 (3.53%) | 157 (13.27%) |
| 2. Aβ+/Aβ- in CN + MCI | 68 (41.72%) | 162 (27.00%) | 38 (23.31%) | 314 (52.33%) | 54 (33.13%) | 56 (9.33%) | 3 (1.84%) | 68 (11.33%) |
| **Barcelona** |  |  |  |  |  |  |  |  |
| 1. Aβ+/Aβ- in total cohort | 74 (32.74%) | 469 (39.64%) | 83 (36.73%) | 462 (39.05%) | 57 (25.22%) | 95 (8.03%) | 12 (5.31%) | 157 (13.27%) |
| 2. Aβ+/Aβ- in CN + MCI | 25 (34.25%) | 162 (27.00%) | 25 (34.25%) | 314 (52.33%) | 12 (16.44%) | 56 (9.33%) | 11 (15.07%) | 68 (11.33%) |
| 3. AD vs FTD | 23 (29.11%) | 302 (63.58%) | 21 (26.58%) | 114 (24.00%) | 35 (44.30%) | 46 (9.68%) | 0 (0.00%) | 13 (2.74%) |
| 4. Controls vs FTD | 33 (44.00%) | 127 (30.68%) | 17 (22.67%) | 225 (54.35%) | 2 (2.67%) | 29 (7.00%) | 23 (30.67%) | 33 (7.97%) |
| 5. AD vs DLB | 20 (21.98%) | 259 (61.23%) | 20 (21.98%) | 62 (14.66%) | 48 (52.75%) | 46 (10.87%) | 3 (3.30%) | 56 (13.24%) |
| 6. Controls vs DLB | 49 (56.32%) | 67 (18.51%) | 15 (17.24%) | 224 (61.88%) | 4 (4.60%) | 30 (8.29%) | 19 (21.84%) | 41 (11.33%) |

*The logistic regression models including the LASSO-selected biomarkers P-tau181, GFAP and age-corrected NfL developed in the Amsterdam Dementia Cohort for the six clinical questions were applied to the external validation cohorts Geneva and Barcelona for the same clinical questions, to compare the agreement true positives (TP), true negatives (TN), false positives (FP) and false negatives (FN). Aβ = amyloid status, CN = cognitively normal, MCI = mild cognitive impairment, FTD = frontotemporal dementia, AD = Alzheimer’s disease, DLB = dementia with Lewy bodies, AUC = area under the curve.*

**
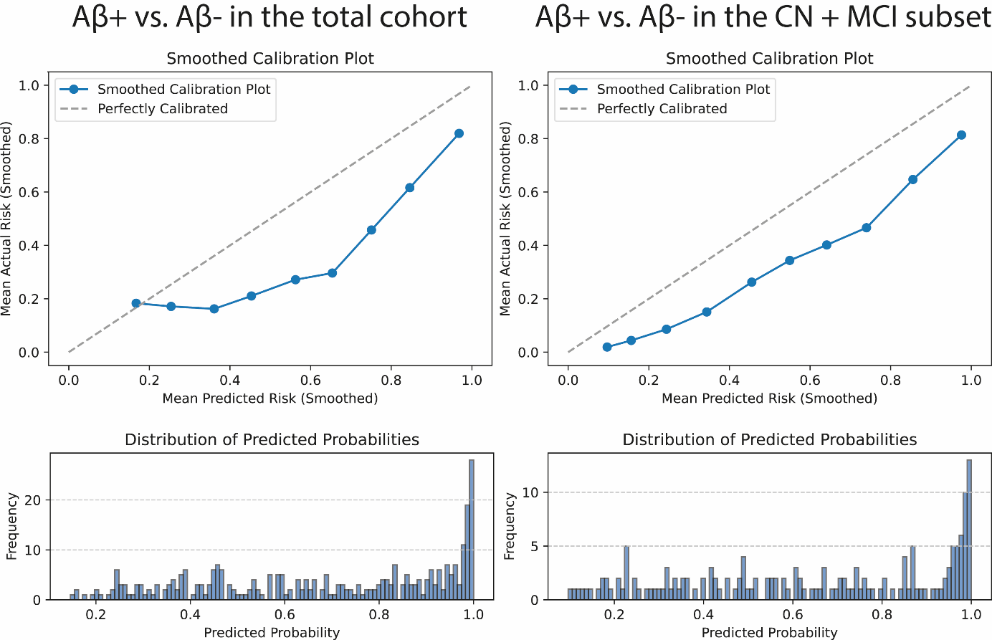

Supplementary figure 3. Geneva calibration plots and the distribution of predicted probabilities for two of the clinical questions.** *The classification logistic regression models including P-tau181, GFAP and age-corrected NfL developed in the Amsterdam Dementia Cohort for two of the six clinical questions were validated for the same questions in the Geneva cohort. Aβ = amyloid status, CN = cognitively normal, MCI = mild cognitive impairment.*


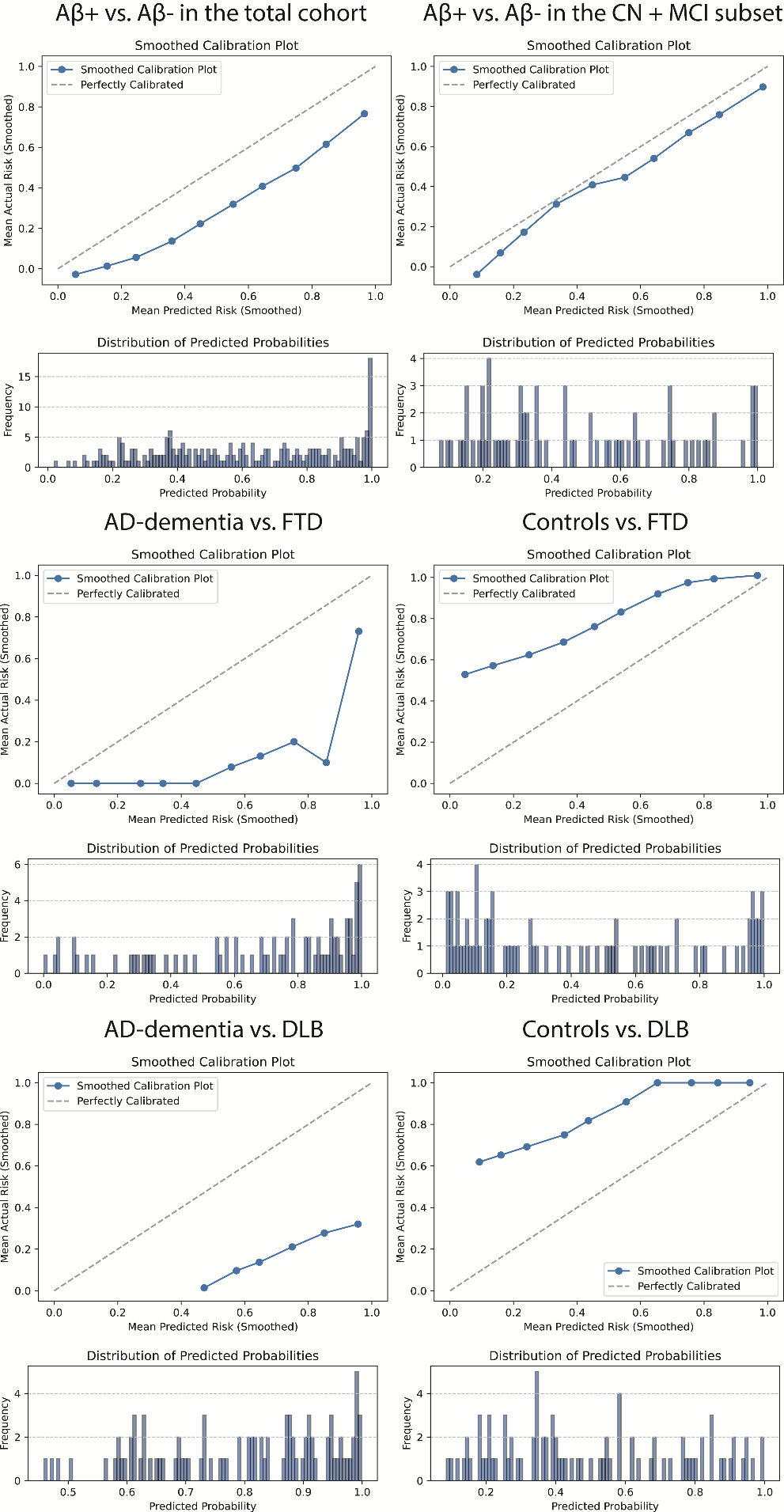

**Supplementary figure 4. Barcelona calibration plots and the distribution of predicted probabilities for the six clinical questions.** *The classification logistic regression models including P-tau181, GFAP and age-corrected NfL developed in the Amsterdam Dementia Cohort for the six clinical questions were validated for the same questions in the Barcelona cohort. Aβ = amyloid status, CN = cognitively normal, MCI = mild cognitive impairment, AD = Alzheimer’s disease, FTD = frontotemporal dementia, DLB = dementia with Lewy bodies.*

**Supplementary table 15. Youden’s thresholds of the individual biomarkers re-calculated in the validation cohorts Geneva and Barcelona in comparison to the Youden’s thresholds in the Amsterdam Dementia Cohort.**

|  | **P-tau181  validation cohort** | **P-tau181 Amsterdam Dementia Cohort** | **GFAP  validation cohort** | **GFAP Amsterdam Dementia Cohort** | **Age-corrected NfL  validation  cohort** | **Age-corrected NfL Amsterdam Dementia Cohort** |
| --- | --- | --- | --- | --- | --- | --- |
| **Geneva** |  |  |  |  |  |  |
| 1. Aβ+/Aβ- in total cohort | 1.80 | 1.81 | 143 | 102 | 0.78 | -0.17 |
| 2. Aβ+/Aβ- in CN + MCI | 1.81 | 1.64 | 143 | 87.2 | 0.79 | -0.17 |
| **Barcelona** |  |  |  |  |  |  |
| 1. Aβ+/Aβ- in total cohort | 1.44 | 1.81 | 114 | 102 | 0.01 | -0.17 |
| 2. Aβ+/Aβ- in CN + MCI | 1.73 | 1.64 | 119 | 87.2 | 0.01 | -0.17 |
| 3. AD vs FTD | 2.90 | 1.71 | 91.9 | 111 | 0.70 | 1.79 |
| 4. Controls vs FTD | 2.02 | 1.28 | 110 | 87.3 | 0.63 | 0.56 |
| 5. AD vs DLB | 2.45 | 1.81 | 208 | 111 | 2.36 | 0.22 |
| 6. Controls vs DLB | 1.57 | 1.70 | 127 | 102 | -0.81 | -0.16 |

*Thresholds at Youden***’***s indices were computed based on ROC analysis over the total data subsets for each cohort separately (Geneva, Barcelona, Amsterdam Dementia Cohort), without internal cross-validation. Thresholds are in pg/mL for P-tau181 and GFAP, whereas they are age-corrected Z-scores for NfL. NfL was corrected for age using a published formula.^46^ Please note that these thresholds are Simoa kit lot dependent, and a value alignment factor is needed to apply the thresholds to new data obtained with other kit lots. Aβ = amyloid status, CN = cognitively normal, MCI = mild cognitive impairment, AD = Alzheimer’s disease, FTD = frontotemporal dementia, DLB = dementia with Lewy bodies, P-tau = phosphorylated tau, GFAP = glial fibrillary acidic protein, NfL = neurofilament light.*
